# Dinosaur Metabolism and the Allometry of Maximum Growth Rate

**DOI:** 10.1371/journal.pone.0163205

**Published:** 2016-11-09

**Authors:** Nathan P. Myhrvold

**Affiliations:** Intellectual Ventures, Bellevue, Washington, United States of America; Perot Museum of Nature and Science, UNITED STATES

## Abstract

The allometry of maximum somatic growth rate has been used in prior studies to classify the metabolic state of both extant vertebrates and dinosaurs. The most recent such studies are reviewed, and their data is reanalyzed. The results of allometric regressions on growth rate are shown to depend on the choice of independent variable; the typical choice used in prior studies introduces a geometric shear transformation that exaggerates the statistical power of the regressions. The maximum growth rates of extant groups are found to have a great deal of overlap, including between groups with endothermic and ectothermic metabolism. Dinosaur growth rates show similar overlap, matching the rates found for mammals, reptiles and fish. The allometric scaling of growth rate with mass is found to have curvature (on a log-log scale) for many groups, contradicting the prevailing view that growth rate allometry follows a simple power law. Reanalysis shows that no correlation between growth rate and basal metabolic rate (BMR) has been demonstrated. These findings drive a conclusion that growth rate allometry studies to date cannot be used to determine dinosaur metabolism as has been previously argued.

## Introduction

In 1978, Case published two classic papers. One studied the evolution of growth rates across the various groups of vertebrates [1]; the other speculated about the growth rates and other life-history parameters of dinosaurs [2]. A key analytical tool in both papers is an allometric regression that uses body mass as the independent variable and the maximum growth rate of body mass as the dependent variable. Case found that various extant groups each have their own scaling relationship of maximum growth rate with body size, and that these parameters plot as well-fit lines on a log-log scale, i.e., the maximum growth rate *G*_max_ = *a M*^*b*^ for constants *a* and *b*.

The realization that dinosaur growth could be measured quantitatively from bone histology [3] led to a body of work that sought to bring this new input into Case’s regression analysis.

These studies employed methods that follow a similar pattern:

1. Use bone histology to estimate the age and size of specific dinosaur specimens, thereby generating a set of age-body mass data points for a given taxon. For extant species, empirical age-body mass points are used.
2. Fit a parametric growth curve to the age-body mass data for each species or taxon.
3. Calculate maximum growth rate *G*_max_ and maximum body mass *M* (or body mass at point of maximum growth rate) from the growth curve for an individual species or taxon.
4. Collect the *G*_max_, *M* data points for species or taxa belonging to a taxonomic group. Estimate the parameters *a* and *b* in the equation *G*_max_ = *a M*^*b*^ by linear regression on log-log transformed data.
5. Plot the *G*_max_ regression lines from many groups, including both endotherms and ectotherms on a log-log scale.
6. Determine the metabolic status of dinosaurs by comparing the *G*_max_ regression line to those of extant groups with known metabolism

In the original study by Case, steps 1–5 were presented for extant groups, because histological analysis of dinosaur growth had not been developed. Instead, Case assumed that dinosaurs followed the growth trajectories of ectotherms, and he speculated on the consequences of them matching extrapolated ectothermic growth allometry.

Subsequently, steps 1–5 were reprised by Erickson and coworkers in a series of papers [4–7] that used histological analysis of dinosaur growth, and data on extant species from Case [1, 2] and Calder [8], without mentioning metabolism or performing step 6. Other authors made similar studies [9].

The first papers to argue directly that dinosaur metabolism can be determined by growth-rate allometry (step 6) [6,10,11], did so without presenting arguments in favor of step 6 other than citations to Case [1, 2] and Calder [8].

Recently two major studies by Werner and Griebeler [12]and Grady and coworkers [13] sought to revisit the Case-Erickson analysis (steps 1–6) with additional data and new statistical and theoretical arguments in favor of a link between maximum-growth-rate allometry and dinosaur metabolism.

Previous work has critically examined the statistical methodology and reproducibility of dinosaur growth-rate studies [14,15]. Many of these criticisms apply directly to the Erickson papers [4,7,10,11,16], which in retrospect were found to have mostly invalid or irreproducible growth-rate results (steps 1–2 above). Some of these issues are relevant to the recent work by Grady et al. and Werner and Griebeler because these newer studies share some dinosaur data sets with the Erickson papers.

However, the focus in the work that I present here is not on the dinosaur growth-data inputs, but rather on much more basic questions that are germane to almost every study in this area stretching all the way back to Case: Why is it is good idea to regress maximum growth rate versus body mass? What can that regression actually tell us about metabolism?

In order to address this, it is important to be explicit about the hypotheses being examined. The key step underlying this entire body of work is step 6, determining metabolism from growth rate allometry. The studies make two distinct hypotheses about how maximum growth rate relates to metabolism.

H1. The metabolism of all members of a taxonomic group is determined by the regression parameters *a* and *b* for the group (from the allometric relationship *G*_max_ = *a M*^*b*^), by comparison with *a*, *b* for groups with known metabolism.
H2. The basal metabolic rate (*BMR*) is directly related to maximum growth rate *G*_max_ by an allometric equation *BMR* = *αG*_max_^*β*^ for constants *α* and *β*.

Hypothesis H1 was implicitly introduced in [10,11] without any supporting arguments in its favor other than referencing Case [1, 2] and Calder [8], as if those references provided a basis for the hypothesis. Case is also heavily cited as the originator of this approach by the more recent studies [12,13].

Unfortunately, the references to Case are inappropriate because his papers did not argue the validity of H1 –or even propose it. Instead Case focused on the broad conclusion that endothermic metabolism enabled some species to achieve higher growth rate. Case argued the proposition that across broad taxonomic groups, metabolic rate could imply growth rate allometry. This has no bearing on the converse proposition that growth rate allometry implies metabolism. It is a well-known principle of logic that *p* → *q* does not imply that *q* → *p*; the converse of a true proposition may be either true or false and must be separately justified.

In addition, Case was skeptical about a tight link between metabolic and growth rates, observing:

Although such early investigators as Brody (1945) and Kleiber (1961) assumed that low metabolic rates should be associated with low growth rates, the basis for this assumption is not readily apparent. Why, in fact, should growth rate and metabolic rate vary with body size at roughly the same rate? The answer is not at all obvious. [1]

After discussing pros and cons, Case summed up [1]:

I conclude that an organism's growth rate is not solely determined by its metabolic rate, although the evolutionary achievement of endothermy seems to have resulted in lifting the physiological restraints upon growth rate enough to produce nearly a ten-fold increase over ectothermic growth rates.

In addition to this, I find that the original Case regression is poorly chosen on a basic statistical basis, mainly because the dependent variable contains the independent variable as an explicit factor. The result of this flaw in methodology is to greatly exaggerate the clustering of data points around the regression lines and thus the resulting power of the regressions (for example, by inflating *R*^2^). The method creates the illusion that the regressions have uncovered an important biological phenomenon, when in fact the outcome is all but predetermined by the mathematics. I demonstrate that when such regressions are performed using more appropriate variables, they provide at best weak support for any conclusions. This issue calls into question the wisdom of using the Case method.

Growth-rate allometry is based on regressions of growth rate and body mass, which summarize the central tendency of variation across the group–in other words, they capture the average behavior over the body sizes and growth rates in the group. This makes H1 very problematic from the point of view of either biological or statistical inference. Statisticians refer to this problem as the “ecological fallacy”: the erroneous conclusion that one can infer individual properties from group-wide averages. The ecological fallacy is widely recognized as an invalid form of statistical inference [17–19]. Both growth rate and metabolism are properties of individual species. Why then, should the metabolism of a species depend on a group-wide average (regression), rather than on the growth rate of that species?

Hypothesis H1 is equally problematic from a biological standpoint. There is no biological mechanism for the metabolism of one member of a taxonomic group to depend on other species in the group. If growth rates determine metabolism, then why should the metabolism of a slow-growing mammal be determined by the regression across its distant and faster growing cousins, rather than by its own growth rate?

The biological constraint on H1 is that shared metabolism, like any trait, can come from only two sources: it is either passed through inheritance or independently evolved. Growth-rate allometry explicitly considers the variation of growth rates across species of different body mass. The most recent common ancestor of a group, by definition, has only one growth rate and body size. The allometry of later derived species cannot have been anticipated by a common ancestor; it is explicitly a derived trait.

The definitions of “dinosaur” and “Mesozoic dinosaur” used in the papers by Grady et al. and by Werner and Griebeler are problematic from an evolutionary standpoint. Because they span both ornithischian and saurischian dinosaurs, the most recent common ancestor of the group would be a basal common ancestor of all dinosaurs, likely in the early Triassic. From a biological perspective, the only shared state among the dinosaur taxa studied is inheritance of traits from that ancestor. So at best this exercise can tell us something about the central tendency of variation from that ancestor. That information informs us only about the metabolic status of the studied taxa, not that of its highly derived descendants.

**Fig 1:**
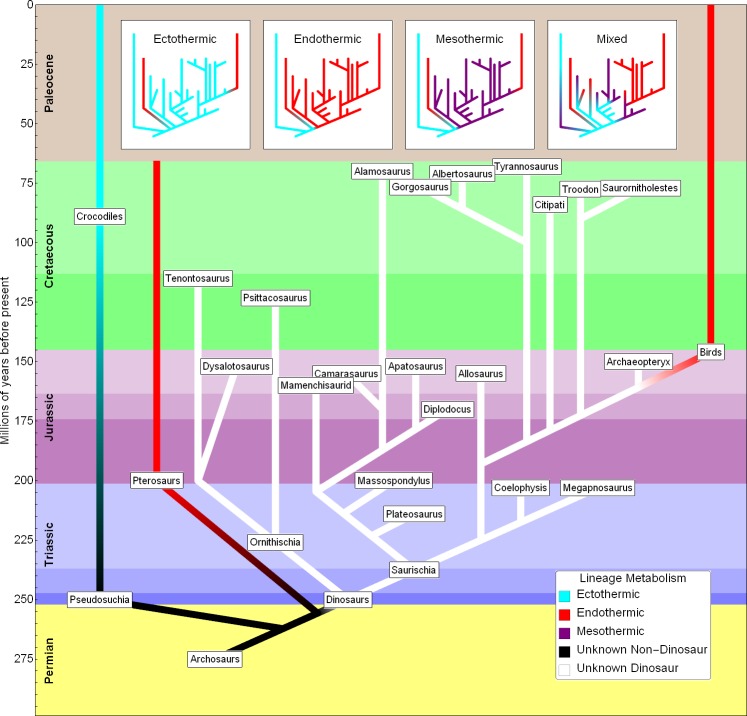
Simplified dinosaur cladogram plotted with approximate ages. Phylogenetic tree data from Grady et al. [13] are supplemented with approximate ages. Lineage metabolism is colored according to the legend. Inset graphs at the top schematically show several hypotheses about dinosaur metabolism.

As seen in the simplified dinosaur cladogram of Fig 1, which shows taxa covered in the papers by Grady et al. and by Werner and Griebeler (based on a tree from Grady et al. supplemented with approximate ages), there are two principle out-groups to dinosaurs: crocodiles and pterosaurs. Crocodiles exist to the present and are ectothermic. Pterosaurs went extinct with dinosaurs at the end of the Mesozoic but which are widely regarded to have been endothermic due to their histology and other factors [20,21].

Birds are the only members of the dinosaur clade that survive to the present day, and they are endothermic. The extant phylogenetic bracket method [22] would suggest on this basis that endothermy might be basal to dinosaurs, or even earlier to the most recent common ancestor of dinosaurs and pterosaurs. This is hardly definitive evidence but is one possibility, shown at the top of Fig 1. Indeed there are even suggestions that endothermy is a basal state for archosaurs [23–26] and that crocodiles are secondarily ectothermic [26].

In contrast, Werner and Griebeler conclude that dinosaurs–including *Archaeopteryx* (see discussion below)–are ectotherms, which implies that endothermy evolved in the clade at some point after birds diverged from their common ancestor with *Archaeopteryx*. Grady et al. hold that all dinosaurs–again including *Archaeopteryx*–have the same metabolism they call mesothermy, again implicitly assuming that endothermy arose within the clade at some point post-*Archaeopteryx* but before modern birds. The metabolic status of Cretaceous birds has not been established, but their ecological and taxonomic similarity to modern birds strongly suggests they, too, are endotherms [27].

There are many other possibilities, however, as illustrated in the top of Fig 1. There is no reason *a priori* to believe that all dinosaurs had the same metabolism. Indeed there are studies that find metabolic diversity [28, 29]. We can surmise that the archosaur ancestor common to dinosaurs and crocodiles was likely ectothermic; we also know that endothermy exists within the clade *Dinosauria*, but apart from that, anything is possible. Various branches of the dinosaur tree may be endothermic or ectothermic or perhaps something else. Indeed, it has been suggested that the largest dinosaurs had a metabolism termed “gigantothermy,” or inertial homeothermy due to their extreme size [29–32]. This illustrates the uncertainty inherent in assuming that a 1 kg *Archaeopteryx* had the same metabolism as a 100 000 kg sauropod.

The situation is more complex still because the inference we are being asked to make is not directly about metabolism but rather about the link between maximum growth rate and metabolism. The wide variation of growth rates within endotherms demonstrates that even if H1 and H2 are correct, there is considerable variation in this relationship. Sauropods, the largest of the dinosaurs, clearly evolved a growth trajectory that allowed them to become the largest animals to walk the earth. Could this unique trajectory alter the relationship between growth and metabolism? In the absence of arguments to the contrary, it seems difficult *a priori* to rule this out.

Each of the extant groups used in these studies spans a range of body sizes and ecological niches, and each evolved from a common ancestor over tens of millions of years of evolutionary radiation and derivation. The evolutionary composition of the extant groups are paraphyletic in some cases, polyphyletic in others. As one example, altricial and precocial birds do not map to clades or evolutionary groups–these behavioral traits have evolved independently across multiple bird lineages [33]. Indeed, it is the behavioral aspect that drives growth, as noted by Ricklefs [34]:

Precocial species, those that hatch with a thick down, able to maintain their body temperatures and feed themselves, grow three to four times more slowly than the young of altricial species, which are highly dependent upon their parents for food and body warmth.

A regression across behavioral groups will, at best, tell us something about the growth rate that is associated with the shared behavior and *not* with the shared lineage. For that reason, we cannot assume that it is legitimate to compare behavior-based groups to lineage-based groups. Indeed the existence of a behavioral trait that has a factor of 3× to 4× influence on growth rate and not on metabolism could potentially confound any purported relationship between growth rate and metabolism.

The reason that H1 focuses on the regression parameters rather than on individual growth rates appears to be based not on principle but instead on the intertwining of the growth rates of individual endotherms with those of ectotherm species, a topic discussed in more detail below.

**Fig 2:**
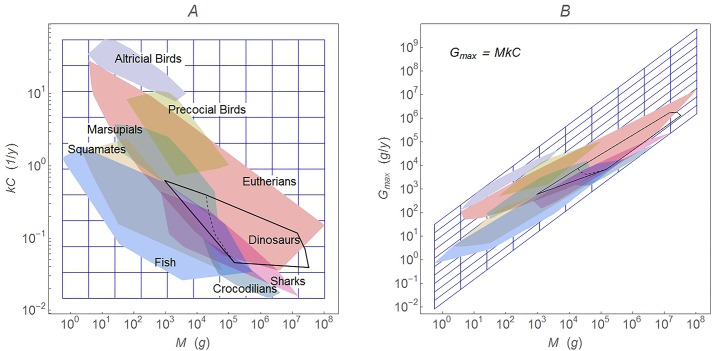
Geometric effect of the shear transformation between log-log transformed *G*_max_ and *kC*. Panel (A) shows regions in the (*M*, *kC*) plane; (B) shows the same regions when transformed to (*M*, *MkC*). The scale is log-log transformed, so the transformation is (log(*M*), log(*k*) + log(*C*)) → (log(*M*), log(*M*) + log(*k*) + log(*C*); see Eq (9).

Werner and Griebeler [12] recognized at least some of the problems with hypothesis H1:

While the averages (regression lines) indicate a clear separation, individual growth rates overlapped between several taxa, even between endotherms and ectotherms (Figs 1 and 2). This indicates that an assignment of a species to ectotherms or endotherms solely based on its growth rate is not possible and that it is inappropriate to apply Case’s or our allometries at the single species level.

Although this observation is correct, application of hypothesis H1 at the group level was not appropriate. No support was provided for the use or validity of regressions at the group level. The fact that group level inferences contradict the conclusion one would draw at the individual species level is a hallmark of the ecological fallacy [17].

Whereas H1 seeks to make broad qualitative comparisons (i.e. endothermic versus ectothermic), H2 looks for a quantitative relationship between *G*_max_ and *BMR*. Grady et al. provide direct metabolic evidence for H1 and H2 by compiling data on *BMR* for many of the species for which they also have growth-rate allometry data and by performing regressions to show a link between *BMR* and *G*_max_. Unfortunately this approach is confounded by the fact that *BMR* and *G*_max_ both have a dependency on body mass *M*. When this spurious confounding factor is removed, their effect disappears.

The issue of averages also applies to H2. If it is interpreted to apply to *G*_max_ at the individual species level, then it shows the same problem as for H1, predicting the wrong *BMR* for many endotherms and ectotherms. If instead it is meant to act only on group-wide regressions for *G*_max_, then the ecological fallacy is in effect.

Both Werner and Griebeler and Grady et al. offer theoretical arguments to support their positions, drawing on the metabolic theory of ecology (MTE) or metabolic scaling theory (MST). Unfortunately, considerable empirical evidence contradicts MTE and MST, particularly with respect to the universality of ¾-power scaling, which is a necessary part of the argument in favor of a link between maximum growth rate and metabolism.

In addition to providing support for these metabolic hypothesis, the two papers provide conclusions about dinosaur metabolism based on their analysis. Unfortunately, they draw incompatible conclusions. Werner and Griebeler conclude that “the growth rates of studied dinosaurs clearly indicate that they had an ectothermic rather than an endothermic metabolic rate [12],” while Grady et al. conclude that that dinosaurs had a unique metabolism, which they term “mesothermy,” intermediate between endothermy and ectothermy [13].

I examine below each of the statistical and biological arguments used by the Werner and Griebeler and Grady et al. studies in favor of H1 and H2. I find that in each case there are strong statistical or biological arguments to refute them.

Growth-rate studies are of great intrinsic interest for detailing ontogeny, but it does not appear that the current argument and evidence is able to make the case that growth-rate allometry has much to say about metabolism. In particular, we cannot use the growth-rate allometry arguments advanced to date to classify dinosaurs as to endothermic or ectothermic.

## Materials and Methods

### Data

The primary data for this study are data points on growth rate (mass versus age) for both dinosaurs and extant groups. These data were obtained from the supplemental material of the most recent studies [12,13] or were provided by their authors.

Grady et al. derived this data from their own growth-curve fits to age–mass data, most of which they obtained by digitizing published graphs. The dinosaur age–mass growth data were obtained directly from Grady, and the curve fits reported by Grady et al. [13] were successfully replicated by using the methods disclosed in the paper. In the course of replicating the fits, however, a number of errors in the dinosaur data sets came to light; these data errors are discussed in S1 Text (see also S1 Fig). I prepared an amended version of the data set (S2 Fig, table in S1 Table) that corrects the errors.

Grady et al. used highly unconventional methods to fit growth curves to their data. For most dinosaur taxa and some extant species, they supplemented the specimen-derived data with hypothetical neonate masses. In addition, for most dinosaur taxa and some extant species, they imposed a constraint that fixed the maximum asymptotic mass to a value chosen from the literature rather than deriving that value from the growth-curve fit. Each of these actions raises serious concerns about the degree to which the resulting growth data is influenced by those assumptions.

A related issue arises from the limited nature of the data, which for most dinosaur data sets span only part of the lifespan [14]. Indeed, only one dinosaur taxon (*Tyrannosaurus rex*) in either the Grady et al. or Werner and Griebeler studies has been shown to have data points that span the age range in which the maximum growth rate occurs. As a result, the maximum growth rate may be an extrapolation far from known data points. Grady et al. supplement the data sets with artificial constraints and hypothetical neonate sizes in part to work around the limitation that the existing data are inadequate to support estimations of maximum growth rates. Because this study focuses on the issue of allometric scaling of growth rate and its interpretation, I nevertheless proceeded with the data derived by Grady et al. using their methodology.

Werner and Griebeler [12] used dinosaur growth data from the literature rather than fitting growth curves themselves. In several cases, they included results that have been shown to be incorrect or irreproducible (see S1 Text), and I thus again produced a corrected data set (S3 Fig, table in S1 Table).

Both the Grady et al. and Werner and Griebeler papers include data for *Archaeopteryx*, which raises more fundamental concerns. Dinosaur growth series are constructed on the basis of lines of arrested growth (LAG), which appear in thin sections of bone. Correspondence with extant examples calibrates the LAGs to be annual markers and thus a standard “clock” for assessing the timing of growth.

However, *Archaeopteryx* bones contain no LAGs. For this taxon, the growth series was instead constructed on the basis of four very uncertain assumptions, as described by Erickson et al. [6]. First, the paper assumes that the bone is laid down at a constant rate. Second, it assumes that we can estimate that constant rate accurately. Together, these first two assumptions mean that *Archaeopteryx* growth is implicitly being modeled as linear growth and that the age of specimens is estimated by dividing the size by that rate. The third assumption is that *Archaeopteryx* growth is ultimately sigmoidal and that all of the known *Archaeopteryx* specimens happen to lie within the linear regime of a sigmoidal growth curve. Fourth and finally, the method assumes that the true sigmoidal curve can be recovered from the linear regime data by imposing asymptotic conditions by fiat.

There is little reason to believe that any of these assumptions are valid. We do not know the correct bone growth rate nor whether it was constant. This is particularly true if *Archaeopteryx* was an ectotherm or mesotherm, as Werner and Griebeler and Grady et al. ultimately conclude, and thus potentially sensitive to environmental variations in temperature. Histological arguments can offer some suggestions, but with very little certainty and very wide bounds. In addition, there is a fundamental contradiction between the assumption that one can model growth as linear for all known specimens and that the growth is ultimately sigmoidal. How does one know that the youngest and oldest specimens observed to date truly lie within the linear regime? We don’t even know that these specimens are “youngest” and “oldest”–all that is known definitively is that they are the smallest or largest described to date.

Moreover, the reasoning that one can use the assumed growth rate to determine the specimen age is highly circular—if the rate is wrong or growth is non-constant, then all of the ages change. Age estimates based on LAG analysis are supported by a tremendous amount of careful research on the bone histology of extant species. It might be possible to calibrate the method used on *Archaeopteryx* by applying it to extant bird specimens of known ages, but that has not been done.

Indeed, if one does suppose that the assumption is correct that *Archaeopteryx* specimens are all in the linear phase, then the maximum growth rate is simply the assumed constant rate. There is little point in making a growth curve, which only obscures the fact that the entire exercise rests on the assumed growth rate. In truth, the data point for *Archaeopteryx* essentially amounts to educated guesses for the value of two parameters: *k* (or equivalently *G*_max_) and *M*. The estimates are, unfortunately, highly correlated because any change to *k* changes the specimen ages and thus the estimate of *M*, assuming that it is determined by regression.

Without a standard clock (such as LAG) to demonstrate otherwise, the most parsimonious assumption is that *Archaeopteryx* grew the way most modern birds grow today, achieving full size in a time frame between a few months (i.e., one growing season) to a year or two [35–37]. If so, then its growth rate would have been similar to that of birds of the same mass.

A different argument is that it is taxonomically inappropriate to classify *Archaeopteryx* with dinosaurs. Most authorities use the term “dinosaur” to mean non-avian dinosaurs, which would exclude *Archaeopteryx*. It is the only avian dinosaur contained within the group that is called “Mesozoic dinosaurs” by Grady et al. and labelled “Dinosaurs” by Werner and Griebeler. In other taxonomic groups, Grady et al. put extinct taxa in with their modern descendants, a policy that would put *Archaeopteryx* in with birds or would mean including modern birds with dinosaurs.

The inclusion of *Archaeopteryx* matters because its body size is much smaller than that of the other members of the group, so the taxa has a disproportionate influence on the regression—and in particular on the value of *R*^2^ and the width of the confidence interval. Because *Archaeopteryx* is a very influential but also very uncertain data point, my calculations on the dinosaur data sets were done both with and without *Archaeopteryx* data.

Data sets for extant species were not checked in the same ways that I analyzed the dinosaur data. The two papers used different definitions of extant groups: for example, Grady et al. split fish into two groups (teleosts and sharks), whereas Werner and Griebeler left fish in one group. I used the same group definitions as in the original papers. Two groups within Grady et al. (monotremeta and testudines) consisted of just two data points each and were omitted from the analysis here.

In addition to growth-rate data, Grady et al. also present metabolic data on basal metabolic rate (*BMR*) for 120 species across both endotherms and ectotherms. These data are potentially quite difficult to compare; they are gathered under differ circumstances, with individual animals that are at different points on their growth–some are juveniles, some are adults. There are important technical differences between *BMR* and metrics like resting metabolic rate (*RMR*). The data for ectotherms have also been adjusted to a standard environmental temperature of 27°C, and the adjustment approach could be questioned. As a result of these issues, the data may be ill-suited to draw like-to-like comparisons across the diverse species in the study. For the purposes of the current work, however, the data were used without attempting to address these issues.

### Statistical Methods

Commercial statistical software (Mathematica v. 10.1) was used to perform the calculations. The models fit to data are presented in S2 Table. Detailed regression results are given in S4 Table and S5 Table.

Grady et al. and Werner and Griebeler used both ordinary-least-squares (OLS) and phylogenetic regressions. Here, OLS regression alone is used because it is sufficient to illustrate the relevant phenomena discussed in the study. The use of phylogenetically independent contrasts (PIC) or phylogenetic generalized least squares (PGLS) would not qualitatively alter the findings because the issues found are based on more fundamental issues, such as the choice of dependent and independent variables.

Werner and Griebeler used a single data point for each extant species but multiple data points for some dinosaur taxa. OLS regressions here are weighted to give the same total weight per taxon as assigned to taxa having a single data point.

## Results

### Growth Rate and Regression

A fundamental goal of the Case studies was to determine how the maximum growth rate of vertebrates scales with their maximum body mass. In the Case paper [1], the maximum growth rate was determined both by curve fitting and by direct observation. Case made a rather unfortunate choice of the dependent variable *G*_max_ for his regressions, which potentially confounds their interpretation, as shown here.

Many growth models are used in biology (see [14] for a list of the most common 75 models). In this case, we wish to model mass as a function of time. Basic dimensional analysis requires that all biological growth models can be written in the form

$$m\left(t\right)=M\;f\left(s\right)+M_{c}.$$ (1)

Where *M* has units of mass, *M*_*c*_ is a constant mass offset, and the function *f*(*s*) is a dimensionless function of a dimensionless variable *s* = *k*(*t*−*t*_*c*_), where the age *t* and the constant offset *t*_*c*_ have dimensions of time, and the growth constant *k* has dimensions of inverse time. For example, for linear growth *f*(*s*) = *s*, whereas exponential growth can be represented by *f*(*s*) = *e*^*s*^. Sigmoidal growth functions can also be put in this form. Logistic growth is

$$f_{\text{logistic}}\left(s\right)=\frac{1}{1+e^{-s}},$$ (2)

and Gompertz and von Bertalanffy growth are given by

$$f_{\text{Gompertz}}\left(s\right)=e^{-e^{-s}},\;f_{\text{vonBertalanffy}}\left(s\right)={\left(1-e^{-s}\right)}^{3}.$$ (3)

In this framework, the maximum growth rate *G*_max_ is given by the chain rule as

$$G_{\text{max}}=\underset{t}{\max}\frac{dm}{dt}=M\;k\;\underset{s}{\max}\frac{df}{ds}=M\;k\;C.$$ (4)

In the case of sigmoidal growth curves, the dimensionless constant factor *C* depends on the specific growth-curve model. For the three most common sigmoidal growth curves:

$$\begin{array}{l} C_{\text{logistic}}={\underset{s}{\max}\frac{df}{ds}|}_{\text{logistic}}=\frac{1}{4}\\ {C_{\text{Gompertz}}=\underset{s}{\max}\frac{df}{ds}|}_{\text{Gompertz}}=\frac{1}{e}\\ {C_{\text{VonBertalanffy}}=\underset{s}{\max}\frac{df}{ds}|}_{\text{VonBertalanffy}}=\frac{4}{9} \end{array}$$ (5)

When we fit a nonlinear growth curve to age-mass data points, we determine both *M* and *k*. To calculate the maximum growth rate, one then multiplies these two variables by *C* to obtain *G*_max_.

Thus all biological growth models have the property that *G*_max_ contains *M* as a factor, and all such models have a growth rate (i.e., an inverse time constant) *k*.

The regression posited by Case used *M* as the independent variable (see discussion below). Regressing *MkC* versus *M* is extremely problematic, however, because the dependent variable contains the independent variable as an explicit factor. The effect of this linkage is to exaggerate the degree of correlation and artificially boost the coefficient of determination *R*^2^, which can create the illusion of a strong biological association between maximum growth rate and body mass. The strong correlation is actually caused by multiplying *kC* by *M* to form the dependent variable.

In mathematical terms, this is equivalent to the hypothesis that

$$G_{\text{max}}=aM^{b},$$ (6)

which can be reformulated using Eq (4) as

$$kC=a\;M^{b-1}.$$ (7)

Thus there is no reason to multiply *kC* by *M* to form *G*_max_; the same hypothesis (6) can equally be tested with (7), a regression on *kC*.

Indeed, regression on the growth parameter *k* versus *M* was done in studies that predate Case [36,38] and these studies were the source for the bird data used by Case. This approach was subsequently used across many species [39].

The biological interpretation of *kC* is the mass-specific maximum growth rate, given by

$$kC=\frac{G_{\text{max}}}{M}=\frac{1}{t_{M}},$$ (8)

where *t*_*M*_ is the time it would take to grow to mass *M* at a constant growth rate equal to *G*_max_. Equivalently, it is the percentage growth per unit time at the peak growth rate.

Of course a real growth trajectory does not stay at the peak rate *G*_max_, so actual growth time is always longer than *t*_*M*_. The growth time from birth to 90% of *M* is typically 1.5× to 2× *t*_*M*_ for many species.

Case used log-log transformed variables, in which case the difference between (7) and (6) is the geometric shear transformation

(x,y)→(x,x+y). (9)

It is a shear transformation that rotates and compresses the (*M*, *kC*) plane along the line *y* = *x*. Fig 2 shows the geometric effect of the shear transformation between *kC* and *G*_max_. This geometric effect acts on the data points and as a result will change any calculation based on the data points, including OLS regression or phylogenetic regressions (PIC or PGLS).

**Fig 3:**
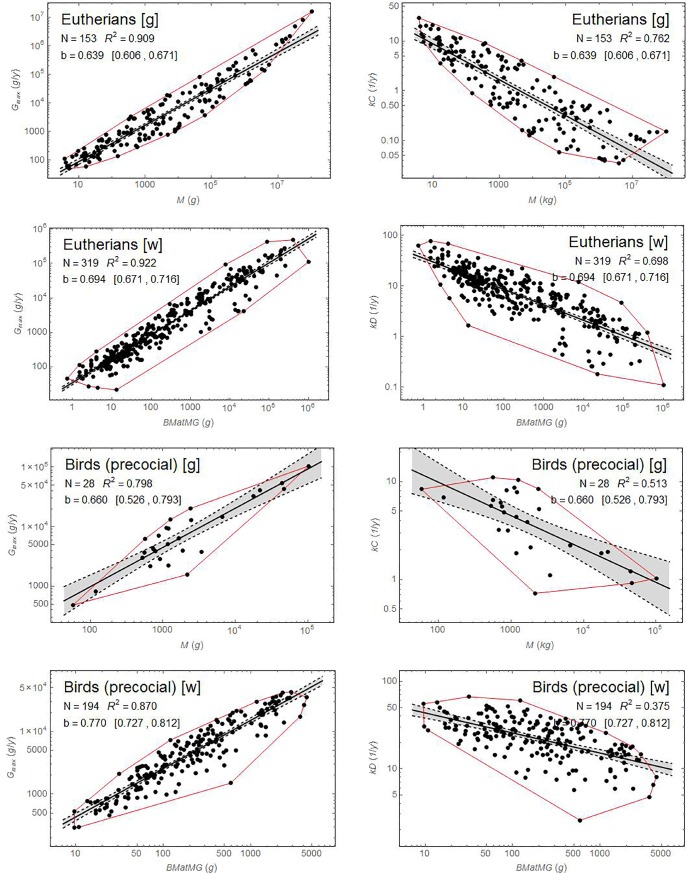
Regression using *G*_max_ or *kC*, *kD* as the dependent variable for several extant groups. Using *G*_max_ instead of *kC* or *kD* reduces the scatter on the graph and greatly increases *R*^2^, while leaving the slope *b* and its 95% confidence interval (in square brackets) unchanged. [w]: Werner and Griebeler [12], [g]: Grady et al. [13]. Red lines delineate the convex hulls encompassing the data points. Shaded regions bounded by dashed lines delineate 95% confidence bands on the regressions.

Fig 3 shows the effect of this transformation for two key extant groups in each study. Note that the estimate of the slope *b* and its 95% confidence interval are not affected by the choice of dependent variable, but the coefficient of determination *R*^2^ is changed, as is the amount of scatter on the plot.

Precocial birds are a key group for both studies. Allometric scaling of growth with mass accounts for only 51% of the variation in *kC* growth rates for precocial birds in Grady et al., and for just 37.5% of the corresponding variation in the Werner and Griebeler study. Corrections for other groups are shown in S4–S9 Figs. The residuals of these regressions are relatively well behaved (S10 and S12 Figs), although for some groups the presence of multiple maxima in the distribution of residuals suggests that there may be some statistical advantage to splitting them into subgroups. Probability plots show that the residuals do not show large deviations from a normal distribution (S11 and S13 Figs).

**Fig 4:**
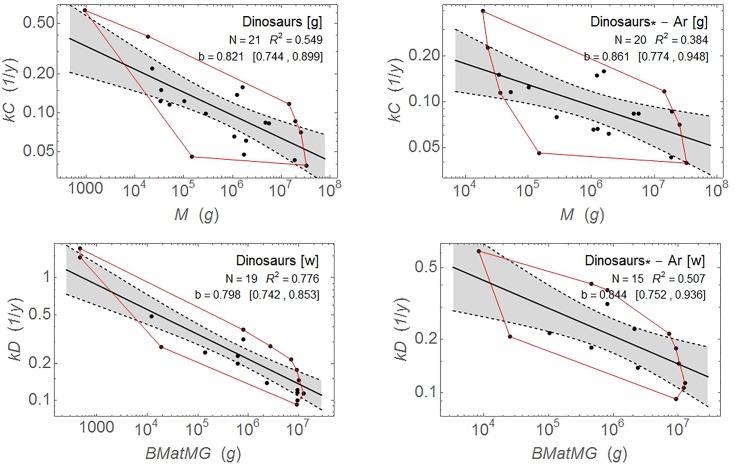
Dinosaur growth-rate allometry. [g]: data from Grady et al. [13]; [w]: data from Werner and Griebeler [12]. Plots on the left are the original data. Plots on the right, labeled *, are the corrected data sets with *Archaeopteryx* removed. Red lines delineate the convex hulls encompassing the data points. Shaded regions bounded by dashed lines delineate 95% confidence bands on the regressions. Note that for the [w] plots, *N* is not the number of taxa because there are multiple data sets for some taxa; the regressions are weighed to account for this. The original data set (upper right) includes 13 taxa; the corrected data set comprises 12 taxa (*Archaeopteryx* is excluded).

Dinosaurs are an even more important group for the current study. Results for the original and corrected dinosaur data sets in both studies are shown in Fig 4. The corrected regressions are quite poor: allometric scaling explains only 51% of the variation in the Werner and Griebeler data and only 38% of the variation in the larger dinosaur data set from Grady et al.

It is often the case that problems with statistics occur in the interpretation of results rather than in the actual statistical calculations, in part because standard statistical software makes the calculations straightforward. In this instance, the value of *R*^2^–and more generally the difference between the variation in the data and the regressions–is important to the interpretation of the results. Case chose regression variables that showed very tight clustering of data points, with high values of *R*^2^, which gives the appearance of being due to a fundamental biological phenomenon. This outcome encourages the use the regression results for classification (and thus hypothesis H1). The smaller the range of variation in the data, the closer the regression results are to actual data. As a practical matter, this relationship would tend to reduce the difference between using the regression versus the actual data.

We now know that the apparently tight clusters are due to the shear transformation (9), not biology. With the correct choice of variables, the effect of allometry explains *only half or less* of the effect for some key groups, so there is a huge amount of scatter in the points. This by itself argues strongly against hypothesis H1. If the allometric regression of maximum growth rate versus body mass has low explanatory power, then why should it be used to predict metabolism?

Grady et al. argue that “self-correlation” between *M* and *G*_max_ is actually a positive aspect of their analysis: “Further, in our analysis, self-correlation emerges not because growth rate and final mass are logically dependent but rather because of the particular manner in which growth rate was calculated” [40]. This is incorrect; the mass-specific growth rate *kC* is a biologically reasonable quantity–it is the growth-rate percentage per unit time at peak growth rate. The peak growth rate in absolute terms (i.e. mass per unit time) has a clear and obvious dependence on mass. It is intuitively clear that is easier for a 1000 kg animal to add 1 kg of new growth in a given time interval than it is for a 1 kg animal to add 1 kg in the same interval. For the former this would represent a 0.1% growth increment, while for the latter it would be 100%, or a doubling of growth. So *G*_max_ is clearly dependent on mass, whereas the mass-specific growth rate *kC* is not.

### Choice of Independent Variable

Grady et al. followed Case [1,2] and Erickson et al. [5–7,10,11,41] in using maximum asymptotic mass *M* as the independent variable. This follows a long tradition of using *M* in this manner in studies of metabolism [42–45] and growth [8,35,46].

Werner and Griebeler took a different approach, arguing that one cannot compare the maximum growth rate from different growth models because it occurs at different ages and masses [12]:

Today, a proper method for estimating growth rates is to fit non-linear growth functions to growth data. The most commonly used growth models describing individual growth are the Logistic, Gompertz or von Bertalanffy growth functions [12–19]. All three functions are similar in shape (sigmoidal), but the location of the point of inflection differs. The von Bertalanffy function has a point of inflection at approximately 30%, the Gompertz function at around 37% and the Logistic function at 50% of asymptotic body mass. Thus, maximum growth, which is observed at the point of inflection and is expressed in absolute maximum growth rate, is not comparable even between these standard models without an appropriate transformation.

This last assertion is not supported by any arguments, and unfortunately is not correct. The value of the maximum growth rate is comparable across all three models because in each case it is defined as the change in mass per time and is given by the first time derivative of the growth curve. In each model, the maxima do occur at different ages and thus different masses, but it is nevertheless statistically and biologically justified to compare them. The concept of fitting a growth curve on a per-species basis is based on the assumption that the growth trajectory–and thus the curve’s parameters (as an approximation)–is encoded in the genome of that species (often called determinate growth, because the properties are pre-determined). In this view, the maximum asymptotic mass of the species *M* is already built into the biological mechanisms regulating growth at all ages, including at the point of maximum growth rate. It thus does not matter that the mass at the time of fastest growth differs from model to model. That is the philosophy behind most authors’ use of *M* as an independent variable for a wide range of natural history parameters.

In effect, Werner and Griebeler present a different biological hypothesis than Case, Erickson, or Grady et al.: viz., that the choice of independent variable matters, and that the choice should be body mass at the age when maximum growth occurs (*BMatMG*). Werner and Griebeler did not discuss their reasoning in detail, nor did they provide a sensitivity analysis assessing the difference this choice makes.

Mathematically,

BMatMG=Md, (10)

where *d* is a constant that depends on the growth model. For example,

$$d_{\text{logistic}}=\frac{1}{2},d_{\text{Gompertz}}=\frac{1}{e},d_{\text{vonBertalanffy}}=\frac{8}{27}.$$ (11)

The effect of using *BMatMG* as the independent variable instead of *M* is to introduce the constant factor *d* on the independent variable, and thus to rescale the dependent variable by the constant *D*:

$$D=\frac{C}{d},\;D_{\text{logistic}}=2,\;D_{\text{Gompertz}}=1,\;D_{\text{vonBertalanffy}}=\frac{3}{2}.$$ (12)

As a result, for the Werner and Griebeler data sets I follow their practice and regress *kD* versus *BMatMG*.

The physical interpretation of *kD* is given by

$$kD=\frac{G_{\text{max}}}{BMatMG}=\frac{1}{t_{BMatMG}}.$$ (13)

Where *t*_*BMatMG*_ is the time it would take to grow from birth to mass *M* at a constant growth rate equal to *G*_max_. As with the interpretation of *kC*, actual growth time is longer than *t*_*BMatMG*_. The growth time from birth to mass *BMatMG* is typically 2× to 3× *t*_*BMatMG*_ for many species.

If *BMatMG* were to be adopted for the Grady et al. study, it would have no practical effect because all of the data points in that study were derived by using a Gompertz growth model. Using *BMatMG* would rescale the x and y coordinates, but since the growth-rate regression is done with log-transformed data, the rescaling would amount only to translating the points. The slope of each regression would be unchanged, and the intercept would be adjusted by an additive constant. Conversely, if the Werner and Griebeler study were to be redone using *M* as the independent variable, some points would move relative to each other, which would change some of the regression results. Unfortunately, the published data set does not specify which model is used for each extant species, so I was unable to test the size of this effect.

### Intragroup Variation and the Ecological Fallacy

It is common in statistics to compare multiple groups by estimating a statistic, such as the mean, for each group. The larger the sample of the group, the better the estimate of the statistic in question. With sufficiently large samples, one can get sharp estimates of the mean mass of each group, and these means can be quite distinct even if there is considerable overlap among the groups. As an example, the Centers for Disease Control and Prevention’s National Health and Nutrition Examination Survey, in its 2013–2014 data release [47], measured the mass of male and female humans age 20 or older, with the result that the mean male mass was 83. 1 kg and the mean female mass was 72.0 kg, a difference of 11.1 kg.

One could summarize this by noting that, on average, men are heavier than women, but this does *not* imply that we can infer that men are heavier on an *individual* basis–i.e. that all men are heavier than all women. The conflict between conclusions drawn on the group average and at the individual level is known as the ecological fallacy.

The original Case studies arguably did not fall into the ecological fallacy because Case was interested in what could be termed the “forward inference” that different groups of animals had different allometry. Grady et al. and Werner and Griebeler do fall into the ecological fallacy because they seek to make the reverse inference–i.e. to classify dinosaur metabolism by comparing their mean growth-rate allometry to that of extant groups.

Returning to the example of human body mass cited above, we cannot use the mean body mass to classify individuals as male or female by their body mass with any reasonable accuracy. Given a group of *N* adult humans, *all of them the same gender*, we could do the classification if *N* is large enough. Simple bootstrap calculations on the human body mass data set cited above show that one needs *N* ≥ 67 to do the classification to the 95% confidence level. Note that the classification, in effect, applies the group average to the individual (classification as male or female)–this is valid *only* because we assume up front that everyone in this group is of the same gender.

That is a very important limitation. If instead one permits three kinds of group–all male, all female, or randomly male and female with equal probability–then the group size for 95% confidence level jumps to *N* ≥ 303. If we chose more groups with known *a priori* odds of the male/female mix, *N* becomes bigger still, and if the *a priori* odds of group composition by gender are unknown, the classification problem becomes effectively impossible for any *N*. That is the ecological fallacy in action: with sufficiently strong constraints (such as certain knowledge that all members of a group are the same gender and that *N* is large enough), we can make a valid (although still statistical) inference. But without those strong constraints, we cannot do so.

The relevance to the Grady et al. and Werner and Griebeler studies is that we explicitly do *not* know that all dinosaurs had the same metabolism. This is particularly true because their dinosaur data sets span the range from basal dinosaurs to highly derived taxa over an interval of more than 100 million years. It is thus analogous to the case of classification with unknown gender ratio. Even if we suppose that the limited data set available gives us a true estimate of the average dinosaur growth-rate allometry, there is no way to use that to classify all dinosaurs because they could have different metabolisms (see inset graphs in Fig 1).

A second point is that there is a clear and obvious biological meaning to the group average of body mass for male and female humans. We know there is a strong genetic difference between men and women involving the X and Y chromosomes. So the biological meaning of the mean mass is presumably the signal due to that chromosomal difference.

But the group average across the Grady et al. or Werner and Griebeler groups has very uncertain biological meaning, as discussed above. An average across a monophyletic group (supplemented with high-quality, time-resolved phylogenetic data) could provide the allometry of the most recent common ancestor. That could *not* be used to classify the individual members of the group, however–*it would apply only to classifying the ancestor*.

Since the fossil record has not provided unambiguous, time-resolved phylogeny for all dinosaurs, it is not possible to say with certainty that the phylogenetic regression methods used in [12,13] actually do recover the common ancestor of all dinosaurs. But even if they do, that would be irrelevant for classifying the derived taxa from that ancestor. Instead we have a mean allometry across non-uniformly sampled taxa spanning almost 200 million years. It is far from clear what this means biologically.

**Fig 5:**
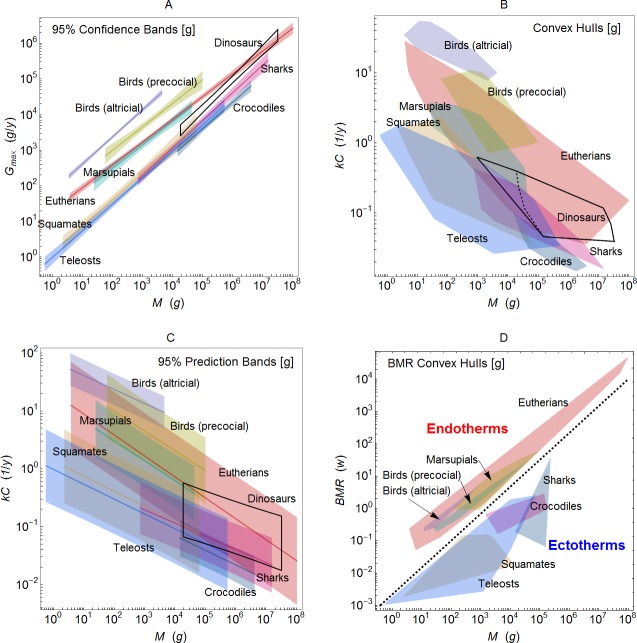
Confidence and predictions bands versus convex hull. Plot (A) shows the results of regression on *G*_max_ for data sets from Grady et al. [13]. Shaded regions are the 95% confidence bands for the regressions. Plots (B) shows the range of variation within each group as a shaded convex hull polygons containing the data from each of the two studies, respectively. Dinosaurs are shown as a black outline; the dashed black outline represents dinosaurs excluding *Archaeopteryx*. Plot (C) shows the single prediction bands for regression of *kC* versus *M* for each group. Plot (D) shows the convex hull polygons for mass and *BMR* data from [13].

Dinosaurs are not the only problem. The groups used in the Grady et al. and Werner and Griebeler studies for extant animals do not have a consistent theme. For altricial and precocial birds the theme is behavioral at best, as discussed above. Other groups are monophyletic, paraphyletic, or polyphyletic, so the biological interpretation of the mean allometry is unclear. Grady et al. and Werner and Griebeler both implicitly assume that a different mean allometry for the group can be interpreted as being due to metabolic differences, but that is far from clear. Altricial and precocial birds do differ in metabolic ways, but also in mass, as can be seen in the convex hull plots of Fig 5B and S23 Fig. Which effect is more important? One cannot tell because the effects of larger body mass may confound the analysis of growth allometry.

Indeed if one took the group of eutherian mammals and arbitrarily divided it into two groups above and below a certain growth rate threshold, the difference between the resulting groups would look much like the difference between altricial and precocial birds. Such a division would highlight different mean allometry, but that does not mean there is a true biological basis for it.

The human body mass analogy above is scalar, or one-dimensional. Any approach to using maximum growth rate to determine metabolism must confront a basic issue: species have both a mass *M* and a maximum growth rate, which could be expressed as *G*_max_ or *kC* or *kD*. Thus a species is fundamentally represented by a point on a two-dimensional mass vs. growth rate plane rather than by a single scalar quantity. As an example, what is the appropriate way to compare the growth rates of two species, one that has a mass of 1 g and the other of 10^6^ g? Similarly, if one measures metabolism directly via BMR, one also has a two-dimensional problem because a 1 g and a 10^6^ g animal will have vastly different total power outputs in watts.

This issue is one reason to apply regression. By understanding how growth rate scales with maximum body mass, we can reason about species across a range of sizes. Regression is entirely analogous to the mean in a scalar example like body mass–it measures the central tendency of the group, but it yields two parameters (in this case) rather than one. The issues with the ecological fallacy and group definition all apply; the only difference is that we must compare two parameters rather than one.

Fig 5A shows regression results for Grady et al. Each paper [1,5,7,9–13,16] includes a plot of this kind (step 5), either with or without 95% confidence bands. The key message that these plots convey is that groups have clearly separate regression lines that for the most part do not overlap. Endotherms and ectotherms show quite different growth-rate allometry in these plots. The visual appeal of these plots due to clearly separated regression lines with narrow 95% confidence bands is a principal reason that the results of prior studies seemed compelling. Qualitatively similar plots can be made for data from the Werner and Griebeler data (S23 Fig).

Unfortunately this picture is very misleading, as can be seen in Fig 5C, which plots the convex hull (smallest enclosing convex polygon) of *kC* versus *M* of the data points for each group. The convex hull plot shows that there is considerable overlap in the growth rate allometry of individual species; it is only the group-wide averages that are distinct.

The conceptual error hiding within Fig 5A is that the 95% confidence bands reflect the constraints on the group central tendency under regression. The tight bounds on the eutherian mammal group tell us that the average allometry across 153 extant mammals is well constrained. Biologically, it is unclear what interpretation should be applied to the average eutherian allometry, but in any event it is not the appropriate statistical method for classification of individuals. The convex hull delineates the total range of variations across individuals in each group. The appropriate statistical measure of the individual variation, and thus classification, is given by the single prediction band (also with 95% confidence) which is shown in Fig 5C. The difference between these two is, in effect, the ecological paradox in action. While the group mean allometry might be tightly constrained, the variation within the group is not.

Rather than being cleanly separated, the growth data for each group overlap considerably. Overlap of this kind also exists in a plot of *M* and *G*_max_, but the shear transformation (9) tends to hide the overlap in a tight cluster of points (Fig 2 and S15 Fig). The original regressions of Case [1, 2] had the compelling property that they seemed to separate disparate groups into distinct bands (i.e. Fig 4A and 4B). This is not true for the individual species-level data, however.

Note that this is *not* the case for direct measures of metabolism. Fig 5D shows the *BMR* versus *M* data of Grady et al., plotted as convex hulls. There is a clear separation between endotherms and ectotherms, even at the individual species level. Note that BMR here is in watts, and it thus depends on the total mass of the animal. One can also look at mass-specific metabolism *BMR*/*M*–i.e., watts per gram of body mass, which is analogous to *kC*. That metric, too, can cleanly separate endotherms from ectotherms (S14 Fig). This poses a major challenge for hypotheses H1 and H2. Maximum growth rate and metabolism are both properties possessed by individual species and presumably encoded in their genomes. If the two parameters are closely tied, as the hypotheses hold, then why is it that groups can be cleanly separated by metabolism, but not by maximum growth rates?

The overlap in the species-level growth-rate and mass data can be demonstrated in other ways as well. Tables 1 and 2 quantify the overlap by showing a count of the data points in each group that fall within the convex hulls. As an example, data on the eutherian mammals from Grady et al. includes members that fall within the convex hull of every other group except altricial birds. In data from Werner and Griebeler, the eutherians include members that overlap the convex hull of every other group.

**Table 1:** Data points that lie inside the convex hull polygon of each group. The data points from Grady et al. [13] arranged vertically and the convex hull associated with each group horizontally. Because there is substantial overlap among the convex hulls, a data point may belong to more than one convex hull.

		Data points								
		Birds (altricial)	Birds (precocial)	Eutherians	Marsupials	Dinosaurs	Crocodiles	Squamates	Sharks	Teleosts
Convex hull	Birds (altricial)	35	3	0	0	0	0	0	0	0
	Birds (precocial)	2	28	32	10	0	0	0	0	0
	Eutherians	1	21	153	18	17	0	5	13	11
	Marsupials	0	3	26	19	3	0	1	1	2
	Dinosaurs	0	0	20	3	21	0	0	11	6
	Crocodiles	0	0	1	0	1	12	1	3	1
	Squamates	0	0	11	1	0	2	26	6	37
	Sharks	0	0	21	2	6	3	4	22	20
	Teleosts	0	0	20	2	5	6	23	18	61

**Table 2:** Data points that lie inside the convex hull polygon of each group. The data points from Werner and Griebeler [12] arranged vertically and the convex hull associated with each group horizontally. Because there is substantial overlap among the convex hulls, a data point may belong to more than one convex hull.

		Data points						
		Birds (altricial)	Birds (precocial)	Eutherians	Marsupials	Dinosaurs	Reptiles	Fish
Convex hull	Birds (altricial)	380	183	73	1	0	0	0
	Birds (precocial)	145	194	96	10	0	0	0
	Eutherians	84	132	319	21	7	20	20
	Marsupials	1	5	82	21	0	2	2
	Dinosaurs	0	0	11	1	19	4	3
	Reptiles	0	0	41	2	4	49	103
	Fish	0	0	31	1	5	36	109

Of the 21 dinosaur taxa analyzed by Grady et al., 20 fall within the convex hull of eutherian or marsupial mammals. Conversely, the dinosaur convex hull encompasses more data points for mammals (23) than for dinosaurs. In the data obtained from Werner and Griebeler of the 12 dinosaur taxa, seven dinosaur data points fall within the mammal convex hull, and again there are more data points for mammals within the dinosaur convex hull than there are points for dinosaurs.

Inclusion within the convex hull is only one of many possible ways to measure the overlap. One could instead use the regression lines to make a parallelogram bounded above by the regression line shifted vertically to capture the highest positive residual and bounded below by the regression line shifted to capture the smallest negative residual (S16 Fig). The parallelograms thus generated contain the convex hulls and thus yield even more overlap.

A third, more restrictive overlap classification criterion makes use of distance from a point to the regression line nearest each point (S3 Table). This produces results that are qualitatively similar to those shown in Tables 1 and 2. In the Grady et al. data sets, some points for eutherian mammals are closer to every other regression line (except that of altricial birds) than they are to their own regression line.

Eight of the 21 dinosaur data points are closest to the regression lines of endothermic groups (eutherians, marsupials and precocial birds). The result is much the same when this method is applied to data from the study by Werner and Griebeler: eutherians have at least some members that are closest to every other regression line. Only three of the 19 dinosaur data points lie closest to an endothermic regression line, but the dinosaur regression line is the nearest to at least some members of eutherians, marsupials, reptiles, and fish.

These figures and tables all illustrate an important problem: a plot of maximum growth rate versus mass does not allow clean separation of groups. The seeming simplicity of the regression lines (Fig 5A) arises only because they are averages that show the central tendency of each group. The 95% confidence bands summarize the amount of variation in the central tendency, but do not account for the variation in the underlying data, most of which lie outside the 95% confidence bands.

The regression lines in Fig 5A are distinct, but that is irrelevant from a biological standpoint. The biological question is whether we can determine metabolism for individual species by analyzing the group properties (i.e., the regression parameters *a* and *b*) or the maximum growth rate (*kC* or *kD* or *G*_max_) and mass *M* data points for the individual species.

The various measures of overlap (convex hull, parallelogram, distance to regression lines) tell us that using individual species data to classify metabolism yields different answers than the group-wide averages do.

As an example, suppose that we did not know the metabolic state of eutherian mammals. The regression lines in Fig 5A would classify them with other endotherms (marsupial mammals, birds). The individual species data for eutherians show overlap (Fig 5B and 5C) with both endothermic and ectothermic groups, rendering classification impossible. This would be true regardless of the classification approach used. If instead we look at metabolism directly (Fig 5D and S14 Fig), we see that eutherians have entirely endothermic growth rates that are very easy to distinguish from ectotherms, but entirely overlap other endotherms.

Another issue with averages is their high sensitivity to the sample used. For example, if we choose the subset of eutherian mammals having the lowest growth rate, the average would be classified as an ectotherm. It is only when this subset is averaged together with faster-growing cousins that the regression results predict endothermy. In the case of extant groups, we know the implications of group composition, but it is unclear whether we can have the same confidence with extinct taxa. In particular, we know that there is a fast growing, endothermic subset of dinosaurs–birds. Is it valid to include them? Phylogenetic analysis (i.e. Fig 1) would suggest that it is, but doing so would completely change the group-wide averages.

We could pool all endotherms and all ectotherms together into just two groups, in the hope that this might give the average allometry some valid biological meaning. Perhaps metabolism-specific groups could average out differences due to other factors, leaving metabolism as the only salient difference. Unfortunately, considerable overlap between the two groups remains in both the convex hull and the single prediction bands (S24 Fig).

When group-wide averages give a different answer than individual data, we have precisely the conditions required for the ecological fallacy [17–19]. As discussed in the introduction, there is a strong biological reason to rely on individual data. There is no proposed biological mechanism by which group-wide averages of maximum growth-rate allometry could determine the metabolism of an individual species and override what its own maximum growth rate would dictate.

Grady et al. have argued [40] that convex hulls are an inappropriate tool for evaluation because they “enclose the entire space occupied by a data set” rather than summarizing its statistical properties. They further argued that “Myhrvold’s use of range polygons is misleading in that it implies considerable overlap between taxa that are statistically quite distinct.”

The crux of the issue is indeed determining where data at the species level show considerable overlap and where they are quite distinct (Fig 5B). The groups are not statistically distinct at the individual level–otherwise the single prediction bands would be separated (Fig 5C). Finally, there is no overlap between endotherms and ectotherms in the species-level data for metabolism itself (Fig 5D and S14 Fig). The fact that an average across a group can yield a tight bounds, if the group is of sufficient size, does not imply that this average is very informative about individual group members.

### One Dimensional Comparisons

A different way to approach the two-dimensional comparison problem for growth-rate and mass data is to reduce it to one dimension. Both Grady et al. and Werner and Griebeler used this approach.

Werner and Griebeler performed regressions on their data using an alternate method that fixed the slope of the regression at *b* = 0.75 and used *a* alone to fit the data. Note that regression with one parameter is still an average and thus has the same ecological fallacy issues discussed above for regression with two parameters. The value of 0.75 for *b* has a long history from empirical studies of metabolic scaling [8,42–44] and is predicted theoretically by MTE, as discussed in the next section. Grady et al. perform an equivalent calculation for both *b* = 0.75 and *b* = 0.66, which they refer to as the “mass-independent growth rate”–a misnomer because the parameter does depend explicitly on mass.

The correct interpretation of this approach is that it adjusts the growth rate of an individual species to the growth rate it would have if it had a body mass of 1 g (i.e., log *M* = 0), assuming that the mass dependence is *M*^*b*^. Because the slope *b* is fixed, adjusting for any other value of *M* effectively adds a constant to the distribution. This general method has been explored by others [48].

**Fig 6:**
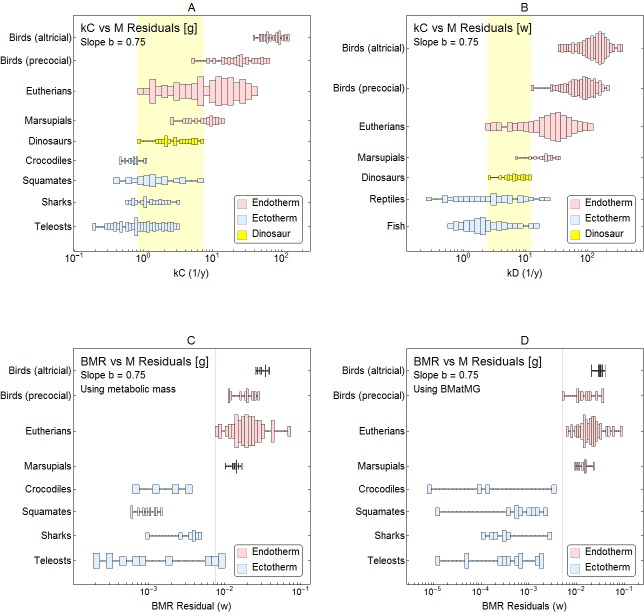
Fixed-slope regression residuals. Each plot shows the histogram of growth rates adjusted to a mass of 1 g, assuming a fixed slope of *b* = 0.75 for data sets from (A) Grady et al. [13] and (B) Werner and Griebeler [12]. Histograms for endothermic groups are shaded light red; those for ectotherms are shaded light blue. The shaded band shows the growth rates that overlap among extant endotherms, ectotherms and dinosaurs (yellow). Plots (C) and (D) show the equivalent fixed-slope regressions for *BMR* data from [13].

Fig 6 shows the results where *b* = 0.75 for both the Grady et al. (Fig 6A) and Werner and Griebeler (Fig 6B) data sets (S25 Fig shows corresponding results where *b* = 0.66). If a clear separation among groups were possible, then one could draw a vertical line dividing the endotherms on the left from the ectotherms on the right. In actuality, the same mass-adjusted growth rate is associated with both endotherms and ectotherms within a wide band (shaded areas of Fig 6).

Fig 6 plots these results in essentially the same way as Werner and Griebeler do in their Fig 2 and Grady et al. do in their S6 and S11 Figs, except using distribution histograms rather than box-and-whisker plots. The overlap pictured in Fig 6 is also evident in their figures.

Instead of using fixed *b* for all groups, one can instead perform the adjustment for each group using a value of *b* determined from the regression for the group. As that value varies group to group, adjusting to a specific value of *M* can yield small differences among groups, but the qualitative picture remains the same: there is no way to separate endotherms and ectotherms at the species level.

One can perform the same analysis, with fixed *b* = 0.75, on basal metabolic rate (*BMR*) versus mass data from Grady et al. (Fig 6C and 6D). In this case, however, a clear separation is possible with little or no overlap between endotherms and ectotherms. Growth rate is very different from *BMR* in that respect, just as it is in Fig 5.

Fig 6 also shows that the groups having the fewest data points (dinosaurs and marsupials) also have the narrowest ranges of residuals, likely due to small sample sizes. As dinosaur data accumulates and the sample size increases, the range for that group will likely overlap even more groups. There is no reason to believe that the small number of dinosaur taxa sampled (20 in Grady et al., 12 in Werner and Griebeler) spans the entire range of maximum growth-rate variation for the clade Dinosauria.

### Theoretical Arguments

Both Grady et al. [13] and Werner and Griebeler [12] offer theoretical explanations based on MST, MTE, and the body of work that has risen around the allometric scaling of *BMR* with mass first discussed by Kleiber in 1932 [42,43]. Kleiber found that *BMR* ∝ *M*^0.75^, a result that set in motion a tremendous amount of empirical and theoretical work in the intervening decades [49–58]. This topic is far too vast to adequately summarize here, but some brief observations will suffice to put it in context.

MTE seeks to explain Kleiber’s exponent 0.75 as a result of the fractal scaling of metabolic networks [58]. This theory generates two fundamental predictions: that the exponents *b* = ±0.75 and in some cases *b* = ±0.25 should be found universally in biology. In particular, the theory holds that *G*_max_ ∝ *M*^0.75^, a relationship of direct relevance here.

Although MTE sparked great theoretical interest, recent work has shown that it is both controversial from a theoretical perspective and apparently refuted by empirical studies [51,58]. The allometry *BMR* ∝ *M*^*b*^ has been reappraised by many studies [49–58]. Some hold that the correct universal exponent is *b* = 0.67, whereas others support a universal *b* = 0.75. Most recent studies indicate, however, that there is no single universal value of *b* and that it instead varies by taxonomic group and by data set. These results cast grave doubt on the applicability of MTE for growth-rate allometry.

Moreover, *BMR* ∝ *M*^*b*^ appears to be only an approximation. Rather than this simple power law, which is a straight line on a log-log plot, the actual relationship has curvature [57–60]. The curvature potentially explains why different studies find different exponents: the exponent varies among data sets that measure different parts of the range.

One can also test for curvature in the regression between maximum growth rate and body mass. Table 3 shows the differences in the corrected Akaike information criterion (ΔAICc) resulting from fitting linear, quadratic, and cubic curves (models in S2 Table) to the *kC* and *kD* growth data. Following conventional practice [61], the best fit is obtained when ΔAIC_c_ = 0. Models are considered to have strong statistical support when ΔAIC_c_ < 2. The regressions are plotted in S18 and S19 Figs.

**Table 3:** Model selection results. The difference between the corrected Akaike information criterion (ΔAIC_c_) was calculated for models fit to log-log transformed *kC* versus *M* data (from Grady et al.) or *kD* versus *BMatMG* data (from Werner and Griebeler), on a group-by-group basis. The models having ΔAIC_c_ = 0 (unshaded cells) are the best fit. Models having ΔAIC_c_ < 2 (bold numbers) have strong statistical support. The results show that substantial curvature effects exist for many groups. Models are defined in S2 Table. Best fits are plotted in S18 and S19 Figs.

		Linear	Quadratic1	Quadratic2	Cubic1	Cubic2	Cubic3	Cubic4
Grady et al. [13]	Birds(altricial)	**1.282**	**0.000**	2.210	5.530	**1.266**	2.844	**0.832**
	Birds(precocial)	**0.119**	**0.000**	2.641	**0.969**	2.502	2.711	4.658
	Eutherians	16.265	73.096	2.011	121.568	**1.457**	**0.000**	**0.835**
	Marsupials	10.808	3.811	2.271	**0.000**	2.630	2.536	5.943
	Dinosaurs	5.596	6.247	7.303	6.846	7.979	7.588	**0.000**
	Crocodiles	**0.000**	**0.798**	3.876	**1.688**	4.151	3.971	8.062
	Squamates	**1.138**	**0.000**	2.752	**0.501**	2.745	2.245	3.890
	Sharks	**0.000**	**0.228**	2.917	**1.548**	3.105	2.847	3.566
	Teleosts	**0.778**	14.577	**0.000**	24.613	5.282	**0.288**	2.340
	*BMR* vs *G*_max_	**0.980**	176.061	**0.000**	143.641	145.539	**1.003**	2.148
	*BMR* vs *kC*	2.012	**1.856**	**0.480**	4.400	3.236	3.316	**0.000**
Werner & Griebeler [12]	Birds (altricial)	52.238	17.152	19.158	52.098	16.979	28.706	**0.000**
	Birds (precocial)	2.870	**0.000**	2.080	**1.558**	2.062	**1.981**	4.044
	Eutherians	**1.928**	34.848	3.597	87.697	18.919	2.880	**0.000**
	Marsupials	**0.963**	**0.000**	2.912	2.615	2.535	3.212	4.176
	Dinosaurs	**0.862**	**0.377**	3.865	**0.000**	3.617	3.694	2.725
	Reptiles	**0.000**	5.943	2.259	12.063	2.771	2.210	4.590
	Fish	**0.000**	16.816	2.145	39.223	2.178	**1.935**	2.475

These results show curvature in growth-rate allometry in many, but not all, groups. In the Grady et al. data sets, the strongest rejection of the linear model (i.e., where the linear model has the highest ΔAIC_c_) is found for eutherian mammals, marsupials, and dinosaurs. For the Werner and Griebeler data sets, in contrast, it is among the altricial and precocial birds where the linear model has the highest ΔAIC_c_. In the case of *BMR*, it is known that there can be a phylogenetic signal and that there is inconsistency among taxonomic groups [62]. That may also be the case for growth rate, in which case phylogenetic regression methods may be more suitable than the OLS method used here.

In studies of *BMR*, quadratic models have been the most commonly used nonlinear models [59,62–64]. For growth-rate allometry, however, cubic models yield the best fits for all of the cases in which the linear model is strongly rejected.

Curvature rules out the simple allometric power laws predicted by MTE. It implies that the effective exponent is a function of mass [59,62–64], a relationship that may be important for dinosaurs, since that group spans an enormous range of body sizes. Indeed, this curvature may be due to factors that would impose an upper bound on body size [59].

Finally, it should be noted that the one-dimensional comparison discussed above and shown in Fig 6 assumes, in effect, that MTE is correct by performing scaling with *b* = 0.75. This still does not help with the fundamental problem, however, which is that the growth rates in Fig 6 overlap.

### Regression of Maximum Growth Rate versus Metabolism

Both Werner and Griebeler [12] and Grady et al. [13] make the argument that scaling properties of BMR and maximum growth rate imply that the two are related. Grady et al., wrote that “Empirical evidence (13) indicates that *G*_max_ scales similarly to *B*, where *G*_max_ = *G*_0_*M*^*α*^. This suggests that *BMR* ∝ *G*_max_ and thus that metabolic rate may be inferred from growth” [13]. (Their reference 13 is the Case paper [1], and *B* is their notation for *BMR*.) Werner and Griebeler similarly noted that “Additionally, similar slopes are observed in regression models of growth rate against body mass or metabolic rate against body mass [5,9,10,12,14,20,37,49,59–61]. Thus a link between growth rate and metabolic rate seems very likely” [12].

This argument is expressed mathematically as follows. Kleiber’s law and MTE focus on the allometric scaling of *BMR* with body mass

$$BMR=a_{1}M^{b_{1}},$$ (14)

and maximum growth rate *G*_max_ also has an allometric scaling law (i.e., Eq 6 above)

$$G_{max}=a_{2}M^{b_{2}}.$$ (15)

Grady et al. use (14) and (15) to relate the two via either of the following direct relationships:

$$BMR=a_{1}a_{2}{}^{-\frac{b_{1}}{b_{2}}}\;G_{\text{max}}{}^{\frac{b_{1}}{b_{2}}}={\alpha G_{max}}^{\beta }$$ (16)

Or

$$BMR=\frac{a_{1}}{a_{2}}\;M^{b_{1}-b_{2}}G_{max}.$$ (17)

Grady et al. argue that (in the notation used here) *b*_1_ = *b*_2_ = 0.75 which makes (16) and (17) become.

$$BMR=\frac{a_{1}}{a_{2}}G_{max}=c\;G_{max}=0.6\;G_{max},$$ (18)

which combines Eqs (1) and (4) of [13]. Eqs (16) and (18) are the statement of hypothesis H2 presented by Grady et al.

This reasoning behind Eqs (14) through (18) is an example of a well-known fallacy in statistical reasoning called the “fallacy of averages” [65–68]. If (14) and (15) were true equalities then this would be correct, but in general they are statistical correlations, which renders (16) and (17) to be incorrect as general statements [65–68].

By using the Grady et al. data set of 120 extant species with both *BMR* and *G*_max_, we can test (18) directly. Unfortunately it fails by large margins, as shown in S22A Fig. Both *BMR* and *G*_max_ are increasing functions of *M*, so we should expect some correlation. However 0.6 *G*_max_ gives an estimate of *BMR* that ranges from a factor of 13.6 too high to a factor of 161.5 too low.

The *BMR* data from Grady et al. was measured from specimens at body mass *M*_*met*_, which is in general less than adult body mass *M*. This could potentially distort the relationship between *BMR* and *G*_max_. To test this, we can make an adjusted *BMR*:

$${BMR}_{adj}=BMR\;{\left(\frac{M}{M_{met}}\right)}^{0.75}.$$ (19)

Note that (19) implies that Kleiber’s law (14) holds during ontogeny. This is related to the assumption by Werner and Griebeler that one must use *BMatMG* rather than *M*–i.e., that the scaling applies at each moment of ontogenetic growth, rather than just the end point. Most studies of Kleiber’s law involve multiple species rather than following the growth of individuals of a single species, so it is far from clear that this assumption is valid. Indeed, intraspecific studies that study ontogenetic scaling have found a different value of the exponent *b* [69–71] than interspecific studies have.

Using *BMR*_adj_ rather than *BMR* does not yield much improvement, as can be seen in S22B Fig; the estimate now ranges from a factor of 17.7 too high to a factor of 7.28 too low. This is a smaller range of variation than without the adjustment, but it still varies overall by a factor of 129. Eq (18) is a rather poor estimator.

Instead of directly testing (18) by using *BMR* and *G*_max_, Grady et al. pursued a regression of what they termed “mass-independent growth rate” as the dependent variable, with “mass-independent metabolic rate” as the independent variable (Fig 2 in ref. [13]). These terms are misnomers: the most natural definition of “mass-independent growth rate” is *kC*, whereas their definition still contains a factor of *M*^0.25^ and thus cannot be “mass-independent.” Their “mass-independent” metabolic rate similarly contains a factor of *M*^−0.75^. It appears to be intended to perform a function analogous to (19).

It is unclear what a regression using these variables has to do with a test of hypothesis H2 or Eqs (16) and (18). In Section III of the supplemental information of [13], Grady et al. present arguments for treating endotherms and ectotherms differently in creating regressions to test the relationship between *BMR* (or *BMR*_adj_) from *G*_max_. They rely on the hypothesis that Eqs (14) and (15) are true at every point in time during ontogeny (i.e., as with (19)), but appear to transform *G*_max_ rather than *BMR* as in (19).

**Fig 7:**
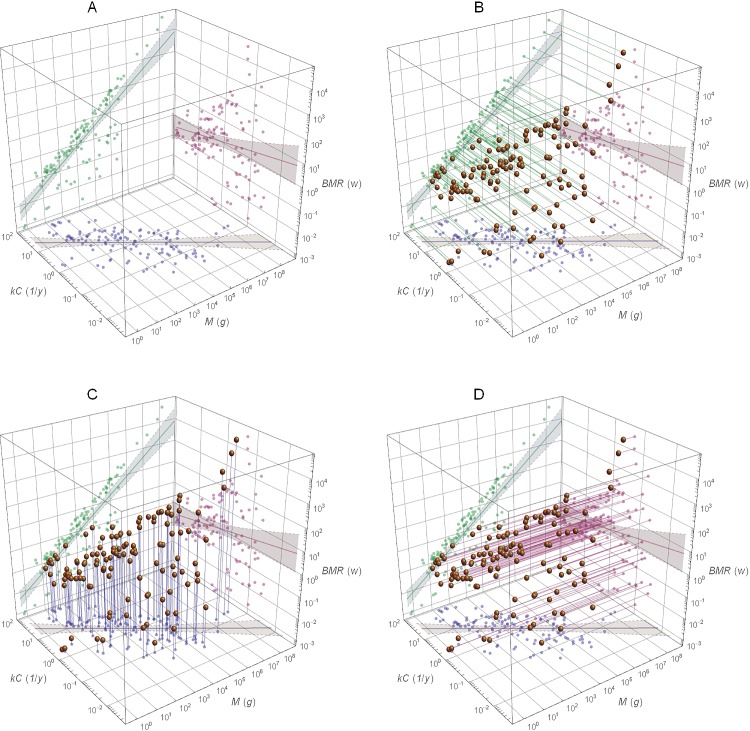
Correlations among *BMR*, *kC*, and *M* are not transitive. (A) shows pairwise correlations between *BMR* and *kC*, *BMR* and *M*, and *kC* and *M* (see also S20 Fig). (B) through (D) show how three-dimensional (*BMR*,*kC*,*M*) points map to each of these pairwise correlations. Even though both *BMR* and *kC* have correlations to *M*, they are essentially not correlated to each other (*R*^2^ = 0.03).

The weak correlation between *BMR* and *G*_max_ shown in S22 Fig is likely due to the fact that they both are increasing functions of *M*. To test this, I regressed *BMR* versus *kC*, which is a genuinely mass-independent variable, as well as versus *M*. The results of these regressions, as well as of *kC* versus *M*, are shown in Fig 7 and detailed in S20 Fig.

I find that there is a moderately strong relationship *kC* ∝ *M*^1−*b*^, *R*^2^ = 0.453 (S20 Fig), and *BMR* ∝ *M*^*b*^, *R*^2^ = 0.622 (S20 Fig). But there is essentially no correlation between *BMR* and *kC*, where *R*^2^ = 0.034 (S20 Fig). If we use *BMR*_adj_ instead of *BMR*, the correlation is slightly stronger but still extremely weak, with *R*^2^ = 0.127.

The fact that we can find regressions between *kC* and *M*, and between *BMR* and *M*–but not between *BMR* and *kC*–may seem surprising. It is a direct refutation of the reasoning in Werner and Griebeler and Grady et al. discussed above in Eqs (14) through (18). This is the fallacy of averages at work [65–68]. The same phenomenon gives rise to the fact that statistical correlation is not transitive [72]. Even though there is statistical support that *kC* ∝ *M*^1−*b*^ and that *BMR* ∝ *M*^*b*^, this does not imply that *BMR* ∝ *kC*^*c*^ for any value of *c*.

S21 Fig plots *BMR* versus *kC* and unpacks the shear transformation, thus separating the convex hulls of the extant groups. This plot can be compared to Fig 2A of Grady et al., in which the choice of variables causes shearing that compresses the range of variation.

The results here cast grave doubt on any relationship between *BMR* and maximum growth rate. A direct test of (18) fails to predict *BMR* (or *BMR*_adj_) from *G*_max_ by a large margin. If instead we use mass specific maximum growth rate *kC* to remove spurious correlation by shared factors of *M*, we find that there is no relationship between *kC* and *BMR* (or *BMR*_adj_), despite the fact that each one is correlated with *M*. Instead of offering proof of hypothesis H2, I find that the data set gathered by Grady et al., fails to show such a relationship, and could even be offered as proof to the contrary. Lovegrove, who used a much larger data set and more sophisticated phylogenetic regression methods than used here, also concluded that growth rate is not correlated with *BMR* for mammals [73].

In response to a previous criticism to this effect [74], Grady et al. argued [40] that it is unsurprising that there is no relationship between *BMR* versus *kC*, but did not explain how that can be consistent with their hypothesis.

## Discussion

Although the Case regressions date back almost four decades, the Werner and Griebeler [12] and Grady et al. [13] studies were the first to attempt to provide concrete evidence of their utility, either generally or in the specific context of evidence for metabolic state. They are to be commended because it is an important issue that was long overdue for a more rigorous treatment.

The results here show that, on balance, the evidence and arguments in favor of hypotheses H1 and H2 are at present inadequate to support them, either as a general matter or specifically for determining the metabolism of dinosaurs.

Hypothesis H1 is particularly difficult to support because it is an instance of the ecological fallacy. There is no statistical or biological reason to use the average (regression) values across a group of species to determine the metabolism of an individual member species rather than using the growth rate for that particular species. This point is not addressed in either the Werner and Griebeler or Grady et al. studies.

Indeed it may not have been recognized as a problem. In response to previous criticism [74], Grady et al. offered several rebuttals [40] that were clearly based on the use of regressions to classify metabolism, without defending or explaining why that should be valid. Case found regressions that tell us about the central tendency of how growth rates scale with body size in different groups. Reversing the logic to determine metabolism by such regressions ignores within-group variation. All mammals are endotherms, but many mammalian species have growth rates lower than those of ectotherms. This is shown clearly in Figs 5 and 6, and S14 Fig, where the plots of growth rate versus mass show considerable overlap among endotherms and ectotherms, while those for metabolism do not. At this level, the issue is very simple–if metabolism depends on growth rate, then why ignore the growth rate of individual species?

No such reason is offered by the studies in question, yet biological and statistical reasoning both agree that one should not ignore species-level growth rates.

One might ask why, if the issue is so simple, it has persisted for so long. Two reasons come to mind. First, hypotheses H1 and H2 were never explicitly called out, nor were they argued and supported. The tacit assumption appears to be that the Case analysis could be inverted and that its converse must be true, although this assumption never seems to have been presented explicitly. Second, statistical arguments were advanced that appear to support the method and to make it a complex statistical matter. Both of these factors tended to obscure the basic biological fact that any link between metabolism and growth rate is tenuous.

On closer examination, the statistical evidence is unfortunately confounded by spurious correlations and multiple fallacies in statistical inference: the ecological fallacy, the fallacy of averages, and the non-transitivity of statistical correlation. As demonstrated above, reformulating the statistical analysis to account for these issues either reduces the explanatory power or removes the effect altogether. Moreover, the data sets used by Werner and Griebeler and Grady et al. do not support hypothesis H1, and the Grady et al. metabolism data set does not support H2.

The theoretical arguments based on MTE or MST have the issue that so far MTE has not been proven empirically to be correct [49–58]. In addition, the results on curvature in growth-rate allometry are difficult to reconcile with the current theoretical arguments.

In addition to the issues presented here, D’Emic [75] criticized the Grady et al. study on the grounds that the estimates of *kC* and *G*_max_ are misestimated for dinosaurs. The LAG data explicitly records periods of very slow or zero growth–the “A” in LAG means “arrested.” Yet as D’Emic points out, the estimates for all such dinosaur LAG analyses [3–5,7,9,10,16,76–78] effectively assume that the duration of that slow-growth period is zero. If instead the period of low growth continued for a substantial fraction of a year (for example, during a resource-poor season, such as winter or a dry season), then the growth rates could be considerably higher–perhaps by a factor of 1.33 to 2, depending on the length of the slow-growth period. This effect would not necessarily apply to the extant animal-growth curves, which are measured directly without the use of LAG. This is a very interesting effect to consider for all future dinosaur-growth analysis, and is orthogonal to the other points made here.

Grady et al. concluded that dinosaurs had a novel “mesothermic” metabolism, intermediate between that of ectotherms and endotherms. This is not standard physiological terminology, and such a metabolic state has not been identified in extant animals. The suggestion begs the question of whether “mesothermy” was a true physiological state of all members of the group or is an illusory state that appears when one averages over a group that contains some ectotherms and some endotherms. Based on the arguments and results presented to date, there does not seem to be a valid basis to support the Grady et al. conclusion of mesothermy among dinosaurs.

Werner and Griebeler [12] offered two alternative conclusions:

Under the assumption that growth rate and metabolic rate are indeed linked, our results suggest two alternative interpretations. Compared to other sauropsids, the growth rates of studied dinosaurs clearly indicate that they had an ectothermic rather than an endothermic metabolic rate. Compared to other vertebrate growth rates, the overall high variability in growth rates of extant groups and the high overlap between individual growth rates of endothermic and ectothermic extant species make it impossible to rule out either of the two thermoregulation strategies for studied dinosaurs.

The study here demonstrates that, Werner and Griebeler’s second conclusion is correct. There is no way to determine dinosaur metabolism because of the high overlap. This is consistent with the finding of Case; there is no evidence for a reliable link between growth rate and metabolic rate. I find Werner and Griebeler’s first alternative–ectothermic metabolisms for dinosaurs–to be unsupported. Restricting the comparison to sauropsids is unwarranted. As Werner and Griebeler point out, we know that some dinosaurs–namely, birds– are endotherms. Either endothermy was an ancestral condition for the clade Dinosauria, or it evolved within the clade (Fig 1). In either case, one should expect a range of metabolic conditions and growth rates, at least some of which are likely to be endothermic.

The data sets used in both papers include both *Massospondylus*, a prosauropod that lived ~200 mya, and *Tyrannosaurus* of 65 mya–a span of 135 million years that runs nearly from the start of the age of dinosaurs to its end. Evolution of growth rates (and perhaps also of metabolism) was shaped by many diverse effects, any of which could easily confound the analysis. There is no reason to believe that all such distant cousins had the same metabolism, nor that an average over them will reflect the metabolism of *all* members of the group. The central tendency may be too high or too low–there is no way to tell.

Dinosaur extinction has heavily edited the range of possibilities in extant species. Limiting the comparison group to extant sauropsids removes any large-bodied endotherms (e.g., mammals) from potential comparison to dinosaurs. Artificially restricting the comparison to sauropsids thus forces a false choice.

Indeed, this example shows the difficulty of assigning biological interpretations to group averages. If we insist on sauropsids as the only points of comparison, we cannot know whether the resulting growth allometry has its properties due to some innate characteristic inherited by all sauropsids, or whether it is confounded by body mass, since the limitation removes all large-mass examples. Is the sauropsid average allometry shaped more by the nature of sauropsids or by the arbitrary restriction to low body mass? It is not possible to tell from the data available.

I find strong support for the second alternative suggested by Werner and Griebeler. Dinosaur growth rates overlap with extant species that span a very wide range of metabolism. So even if there is a link between growth rate and metabolism, one could not say whether dinosaurs are endothermic or ectothermic on the basis of their growth rates. The dinosaur data to date overlap with extant species in both camps.

## Supporting Information

S1 Fig Plot of neonate masses in Grady et al. versus adult mass *M* [13]. Grady et al. used an empirical formula by Dolnik (see S1 Text) to set hypothetical neonate age–mass data points for most taxa, as shown by the solid line. The plot shows that clerical errors led to incorrect values for the labeled taxa. Note that *Citipati* and *Troodon* have directly observed neonate masses, but were also in error.
(JPG)

S2 Fig Corrected versus original dinosaur data points for Grady et al. [13]. The original data points are in black; corrected points are in red. Green arrows show the correspondence. See S1 Text for a description of the corrections.
(JPG)

S3 Fig Corrected versus original dinosaur data points for Werner and Griebeler [12]. The original data points are in black; corrected points are in red. Green arrows show the correspondence. See S1 Text for a description of the corrections.
(JPG)

S4 Fig Comparison of regression on *G*_max_ versus *M* and *kC* versus *M* for crocodiles, dinosaurs, and eutherian mammals in Grady et al. [13]. The red line is the convex hull. The shaded region denotes the 95% confidence band on the regression.
(JPG)

S5 Fig Comparison of regression on *G*_max_ versus *M* and *kC* versus *M* for marsupials and birds in Grady et al. [13]. The red line is the convex hull. The shaded region denotes the 95% confidence band on the regression.
(JPG)

S6 Fig Comparison of regression on *G*_max_ versus *M* and *kC* versus *M* for sharks, squamates, and teleost fish in Grady et al. [13]. The red line is the convex hull. The shaded region denotes the 95% confidence band on the regression.
(JPG)

S7 Fig Comparison of regression on *G*_max_ versus *M* and *kC* versus *M* for extant groups in Werner & Griebeler [12]. The red line is the convex hull. The shaded region denotes the 95% confidence band on the regression.
(JPG)

S8 Fig Comparison of regression on *G*_max_ versus *M* and *kC* versus *M* for marsupials, reptiles, and dinosaurs in Werner & Griebeler [12]. The red line is the convex hull. The shaded region denotes the 95% confidence band on the regression.
(JPG)

S9 Fig Comparison of regression on *G*_max_ versus *M* and *kC* versus *M* for fish in Werner and Griebeler [12]. The red line is the convex hull. The shaded region denotes the 95% confidence band on the regression.
(JPG)

S10 Fig Smoothed distribution plot of residuals for regressions for data sets in Grady et al. [13]. The height of each distribution is scaled proportionate to the square root of the number of samples. Multiple maxima for some groups indicates there may be some structure left in the residuals.
(JPG)

S11 Fig Probability plot of residuals for data sets in Grady et al. [13]. The x-axis is the cumulative probability distribution function (CDF) of the normal distribution, and the y-axis is the CDF of the residual distribution. Normally distributed residuals would lie on the dotted line. The distributions are approximately normal for all groups.
(JPG)

S12 Fig Smoothed distribution plot of regressions for data sets in Werner and Griebeler [12]. The height of each distribution is scaled proportionate to the square root of the number of samples. Multiple maxima for some groups indicates there may be some structure left in the residuals.
(JPG)

S13 Fig Probability plot of residuals for data sets in Werner and Griebeler [12]. The x-axis is the cumulative probability distribution function (CDF) of the normal distribution, and the y-axis is the CDF of the residual distribution. Normally distributed residuals would lie on the dotted line. The distributions are approximately normal for all groups.
(JPG)

S14 Fig BMR/M Convex Hulls. *BMR* versus *M* data from [13] was converted to BMR/M versus M; shaded regions are convex hulls.
(PNG)

S15 Fig Convex hulls for *G*_max_. (A) plots data from Grady et al. [13], (B) from Werner and Griebeler [12]. This figure shows the range of variation using *G*_max_ rather than *kC*, as used in Figs 5B and 4C. The groups are not labeled because they are too close together, but the color coding follows Fig 5 and S16 Fig. Dinosaurs are indicated by the black outline; dinosaurs without *Archaeopteryx* are shown by the dashed black line.
(JPG)

S16 Fig Residual parallelogram plot for data sets. (A) plots data from Grady et al. [13], (B) from Werner and Griebeler [12]. Each parallelogram was formed by taking the regression line for the group; the top boundary is the regression line plus largest positive residual, and the bottom is the regression line plus the most negative residual. The horizontal extent of the parallelogram is determined by the range of the data. Dinosaurs are indicated by the black outline; dinosaurs without *Archaeopteryx* are shown with a dashed black line.
(JPG)

S17 Fig Mass-adjusted growth rates with *b* = 0.66. (A) plots data from Grady et al. [13], (B) from Werner and Griebeler [12]. This figure is the equivalent of Fig 6 but with a different value of the slope parameter *b*.
(JPG)

S18 Fig Best-fit nonlinear model plots for Grady et al. [13] data sets. The best-fit model is plotted in magenta. The 95% confidence band is light gray and bordered by dashed lines. The best-fit linear model is plotted in green, except for groups where the best fit is already linear. See Table 3 for corresponding ΔAICc values.
(JPG)

S19 Fig Best-fit nonlinear model plots for Werner and Griebeler data sets. The best-fit model is plotted in magenta. The 95% confidence band is light gray and bordered by dashed lines. The best-fit linear model is plotted in green, except for groups where the best fit is already linear. See Table 3 for corresponding ΔAICc values.
(JPG)

S20 Fig Correlations among *BMR*, *kC* and *M* are not transitive. (A) plots pairwise correlation between *BMR* and *kC*, (B) between *BMR*_adj_ and *kC*, (C) between *BMR* and *M*, (D) between *BMR*_adj_ and *M*, and (E) between *kC* and *M* (see also S20 Fig). Even though both *BMR* and *kC* have correlations to *M*, they are essentially not correlated to each other (*R*^2^ = 0.03). The situation for *BMR*_adj_ is similar.
(JPG)

S21 Fig Convex hulls of *BMR* versus *kC*. Using these variables, the extant groups from metabolic data of Grady et al. [13] separate cleanly into distinct endothermic and exothermic clusters. The dashed line separates the two groups. The situation is quite different than that shown in Fig 5, where ectotherms and endotherms overlap to a great extent.
(JPG)

S22 Fig Comparison of *BMR* versus *G*_max_. (A) Metabolic data from Grady et al. [13], is plotted on a log-log scale to test the hypothesis that = 0.6 *G*_max_. (B) the same relation is tested with *BMR*_adj_ from Eq (19). Both *BMR* and *G*_max_ are positively correlated with *M*, so we expect some relationship, but the error is very large, as shown by the dashed lines. *BMR* is underestimated by a factor of 13.6 and overestimated by a factor of 161; *BMR*_adj_ does somewhat better, it is underestimated by a factor of 17.7 and overestimated by a factor of 7.3. The grid lines show the region where growth rates for endotherms and ectotherms overlap.
(PNG)

S23 Fig Confidence and prediction bands and convex hulls for Werner and Griebeler [12]. The equivalent of Fig 5 is plotted using the data sets from [12]. Note that there is no plot (D) because [12] does not include mass versus *BMR* data.
(PNG)

S24 Fig Confidence and prediction bands and convex hulls for pooled endotherms and ectotherms. The equivalent of Fig 5 is plotted using data sets for extant animals from [13] after pooling the data into two groups, one containing all endotherms and another containing all ectotherms.
(PNG)

S25 Fig Fixed-slope regression residuals, assuming slope = 0.66. The equivalent of Fig 6 is plotted using a fixed slope *b* = 0.66.
(PNG)

S1 Table Corrected growth-rate data points for dinosaurs. See S1 Text, S1–S3 Figs.
(DOCX)

S2 Table Models used for fitting growth rate to mass. The independent variable *x* is either *log*(*M*) or *log*(*BMatMG*).
(DOCX)

S3 Table Data points closest to each regression line. Table 1 shows the data points from Grady et al.[13] arranged vertically, and the regression line associated with each group horizontally. Every data point is closest to a unique regression line. Table 2 shows data points from Werner and Griebeler [12].
(DOCX)

S4 Table Table of regression results. [g]: data from Grady et al. [13]; [w]: data from Werner and Griebeler [12].
(DOCX)

S5 Table Curvilinear regression results. The models are found in S2 Table, and the identification of the best-fit models is found in Table 3. [g]: data from Grady et al. [13]; [w]: data from Werner and Griebeler [12].
(DOCX)

S1 Text Data Errors in Grady et al. and Werner and Griebeler. (DOCX)
