# Supplementary figures and images for: Dinosaur Metabolism and the Allometry of Maximum Growth Rate
Source: PLoS One. 2016 Nov 9;11(11):e0163205. doi: 10.1371/journal.pone.0163205 (PMC5102473; doi:10.1371/journal.pone.0163205)

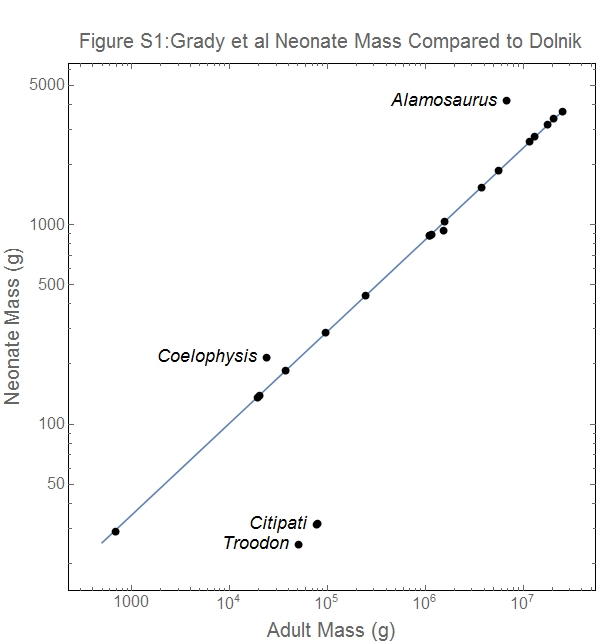

Supplement: S1 Fig — Grady et al. used an empirical formula by Dolnik (see S1 Text) to set hypothetical neonate age–mass data points for most taxa, as shown by the solid line. The plot shows that clerical errors led to incorrect values for the labeled taxa. Note that Citipati and Troodon have directly observed neonate masses, but were also in error. (JPG) [file pone.0163205.s001.jpg]

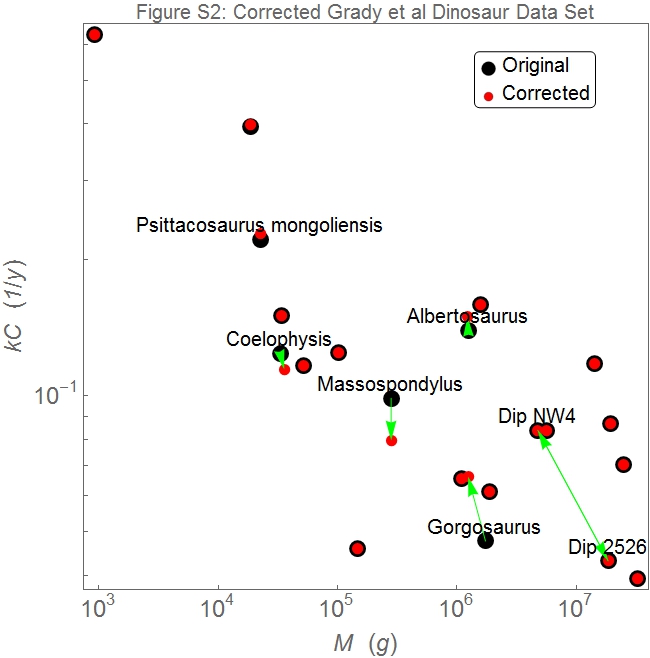

Supplement: S2 Fig — The original data points are in black; corrected points are in red. Green arrows show the correspondence. See S1 Text for a description of the corrections. (JPG) [file pone.0163205.s002.jpg]

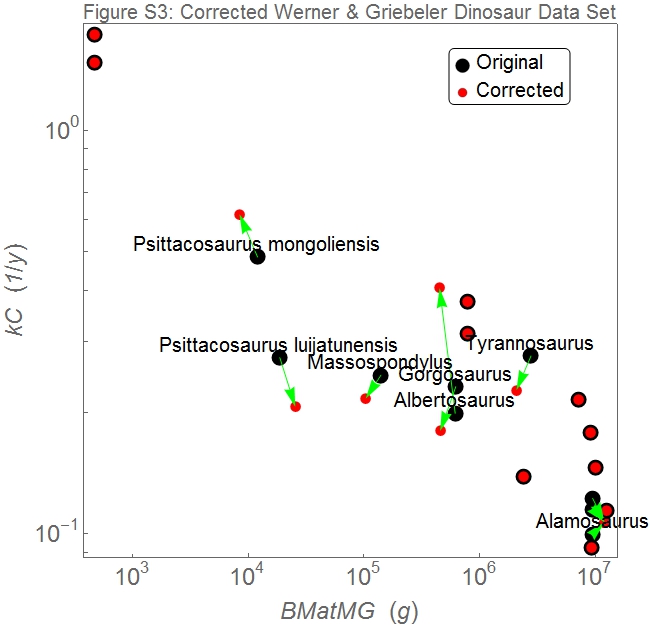

Supplement: S3 Fig — The original data points are in black; corrected points are in red. Green arrows show the correspondence. See S1 Text for a description of the corrections. (JPG) [file pone.0163205.s003.jpg]

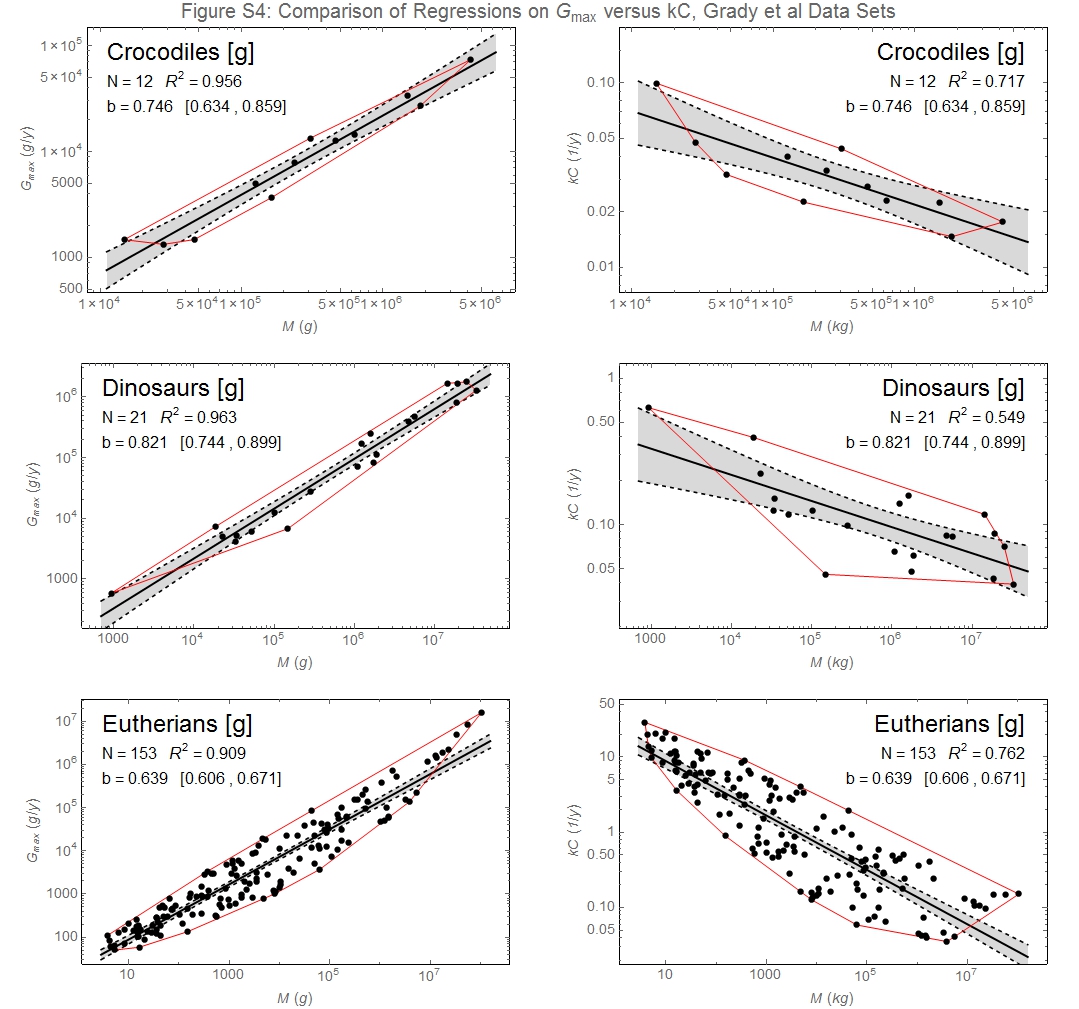

Supplement: S4 Fig — The red line is the convex hull. The shaded region denotes the 95% confidence band on the regression. (JPG) [file pone.0163205.s004.jpg]

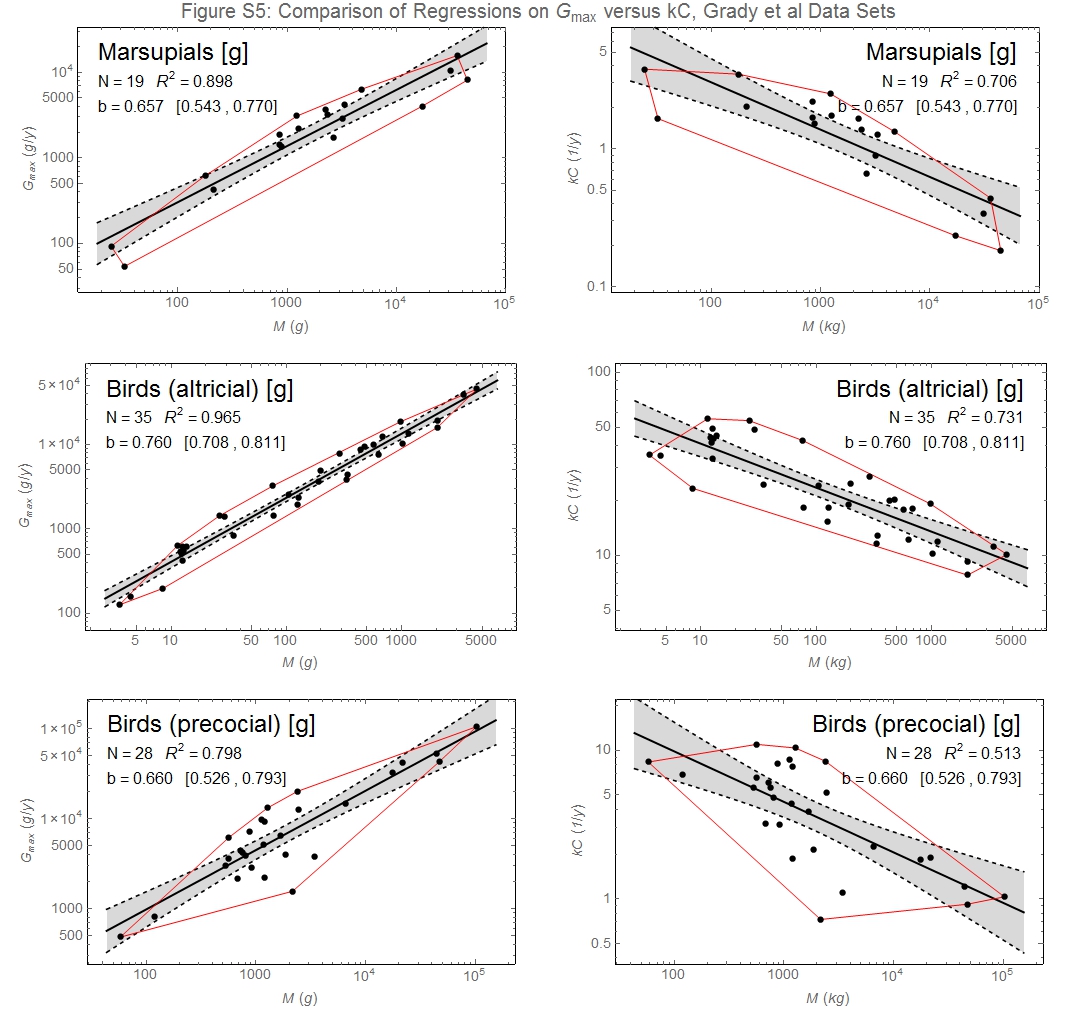

Supplement: S5 Fig — The red line is the convex hull. The shaded region denotes the 95% confidence band on the regression. (JPG) [file pone.0163205.s005.jpg]

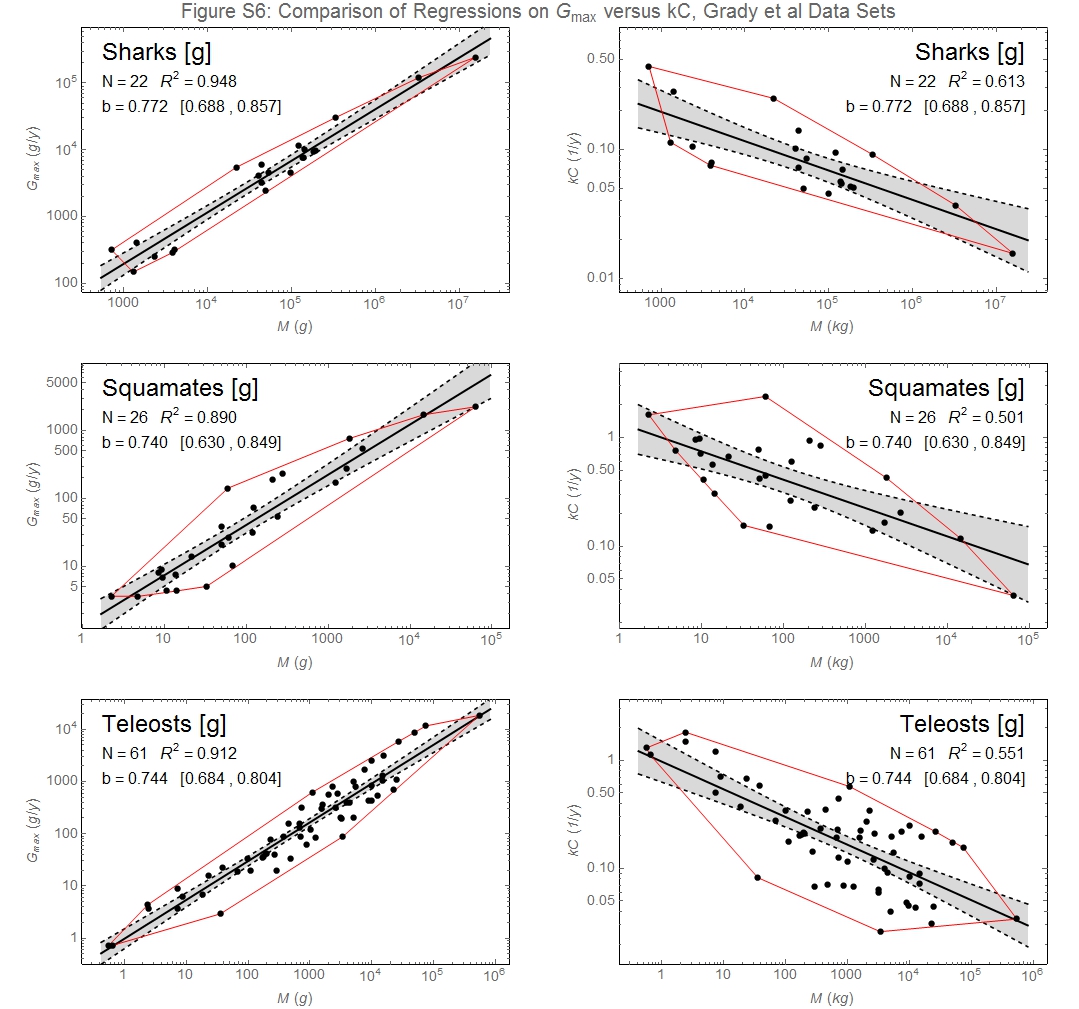

Supplement: S6 Fig — The red line is the convex hull. The shaded region denotes the 95% confidence band on the regression. (JPG) [file pone.0163205.s006.jpg]

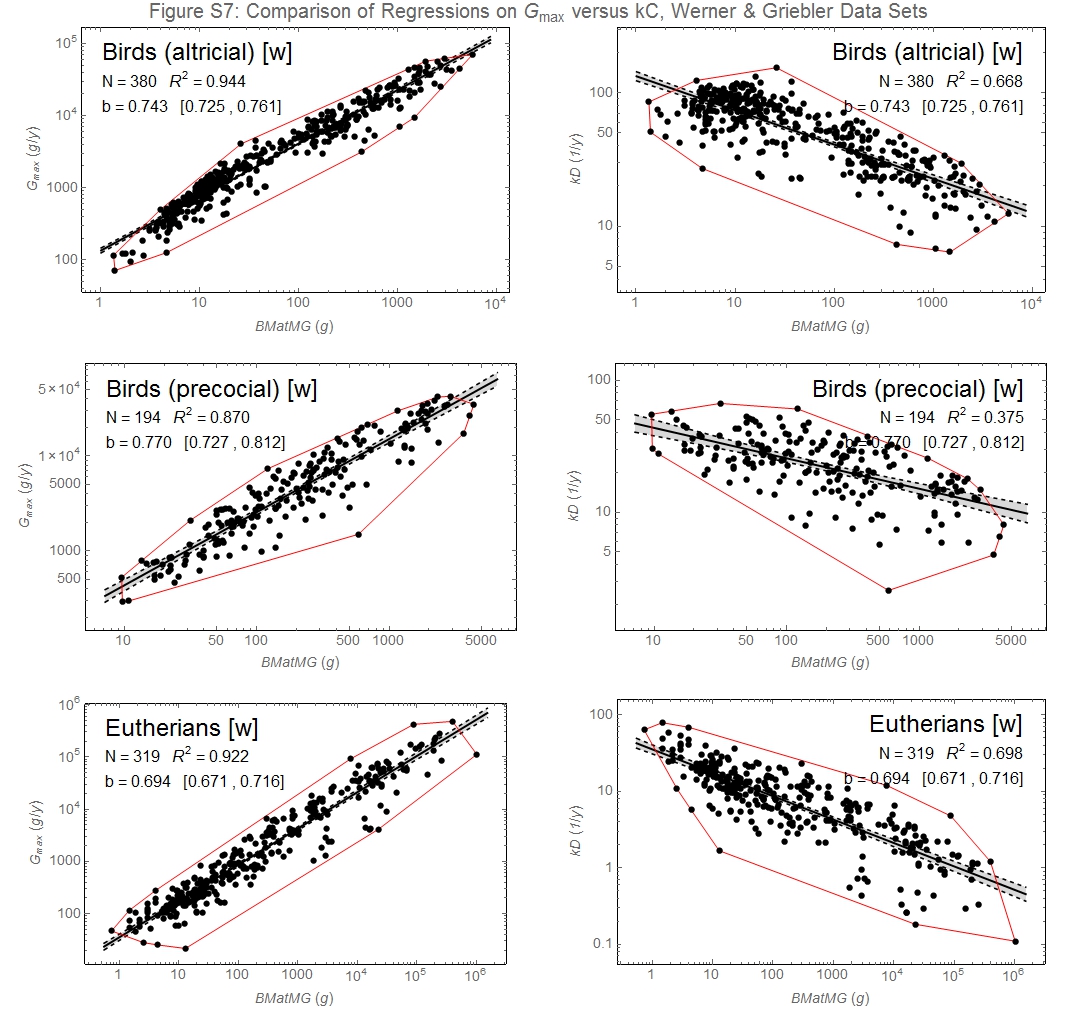

Supplement: S7 Fig — The red line is the convex hull. The shaded region denotes the 95% confidence band on the regression. (JPG) [file pone.0163205.s007.jpg]

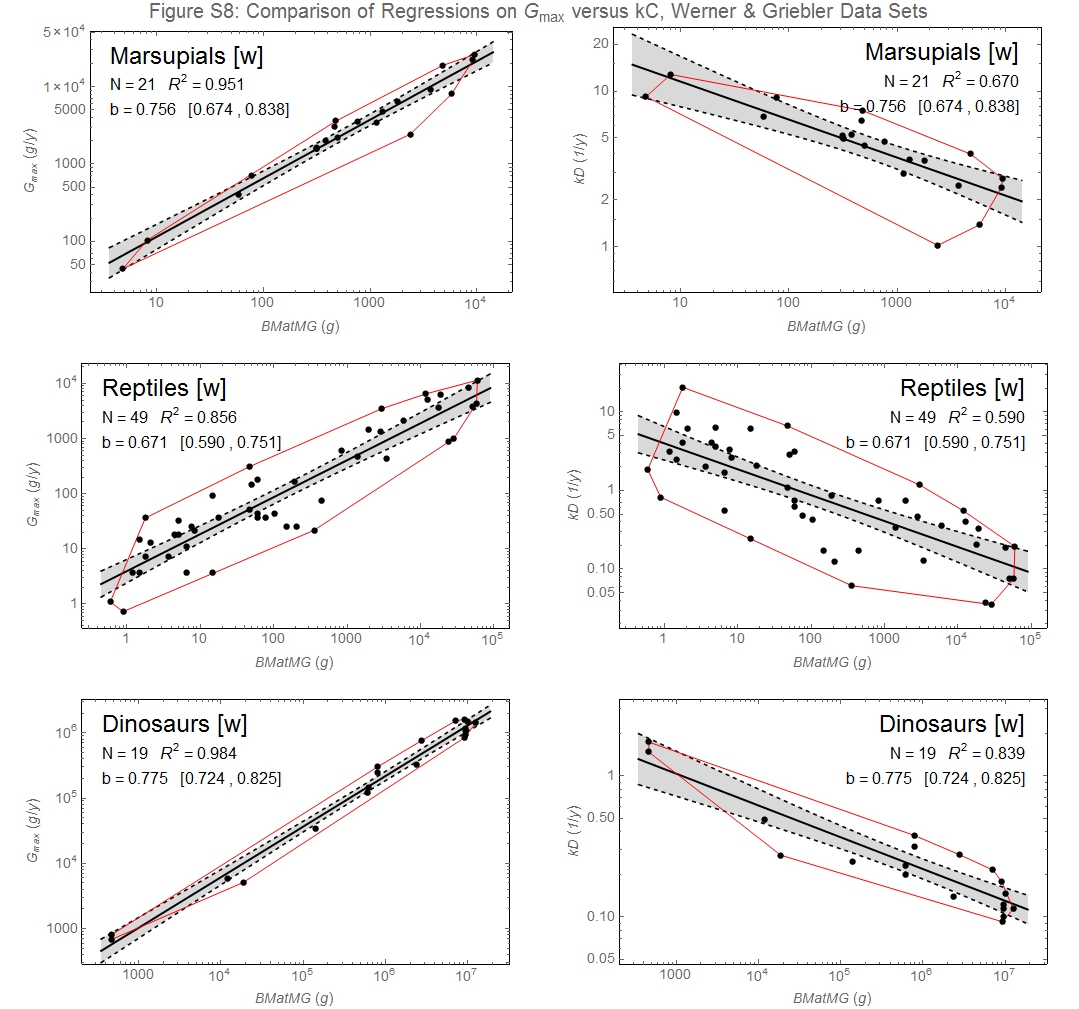

Supplement: S8 Fig — The red line is the convex hull. The shaded region denotes the 95% confidence band on the regression. (JPG) [file pone.0163205.s008.jpg]

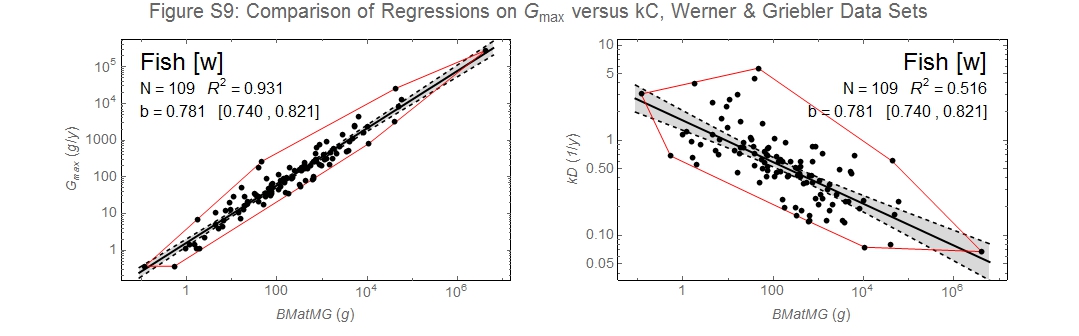

Supplement: S9 Fig — The red line is the convex hull. The shaded region denotes the 95% confidence band on the regression. (JPG) [file pone.0163205.s009.jpg]

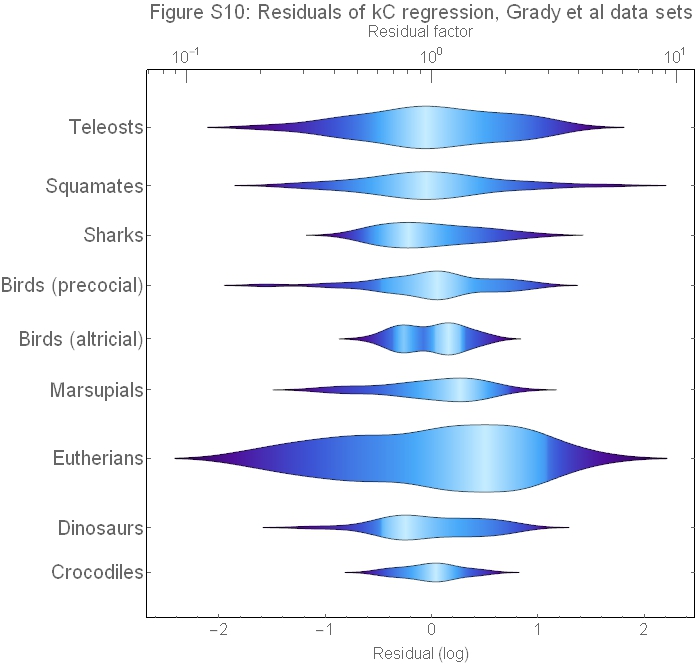

Supplement: S10 Fig — The height of each distribution is scaled proportionate to the square root of the number of samples. Multiple maxima for some groups indicates there may be some structure left in the residuals. (JPG) [file pone.0163205.s010.jpg]

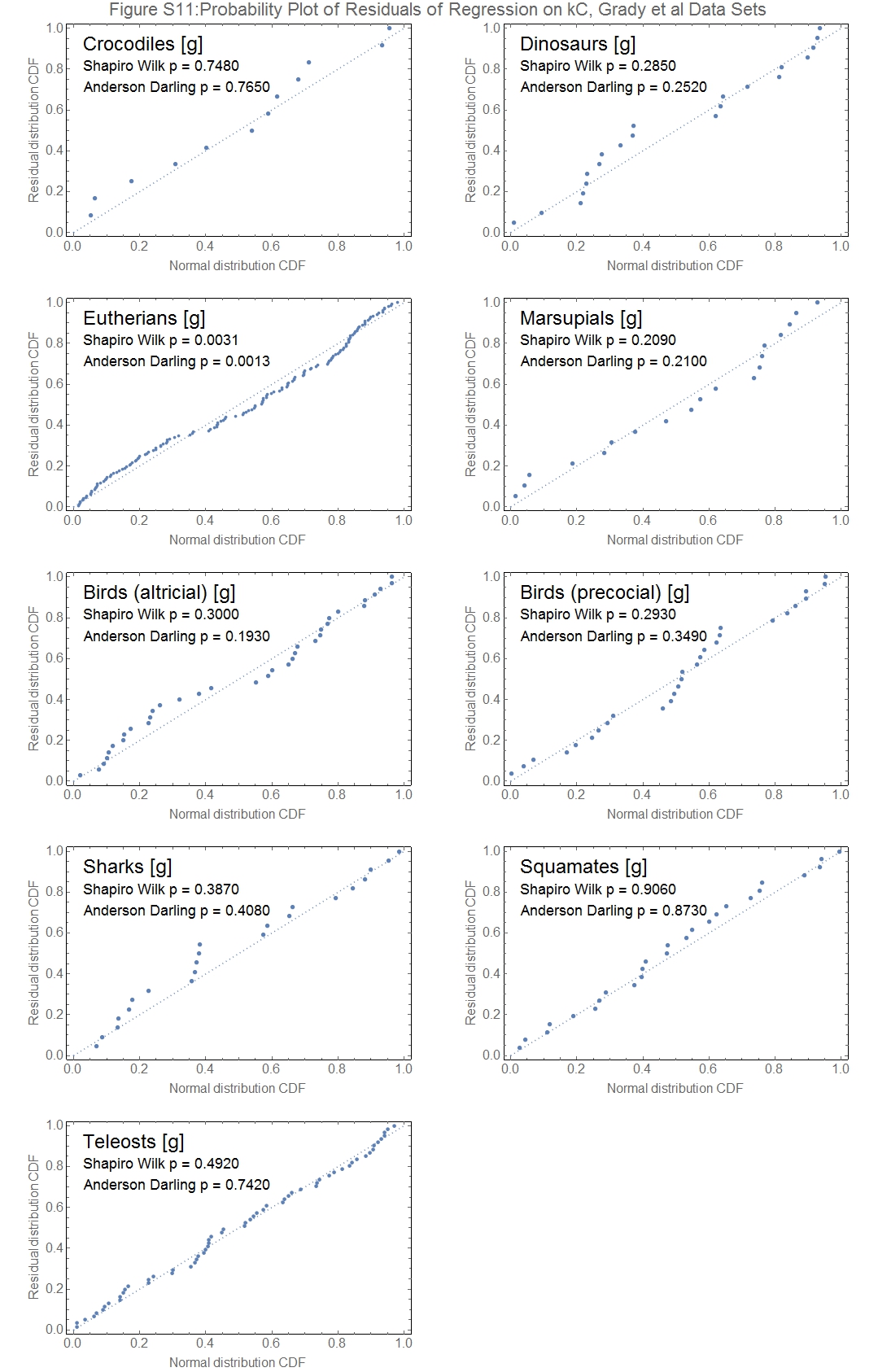

Supplement: S11 Fig — The x-axis is the cumulative probability distribution function (CDF) of the normal distribution, and the y-axis is the CDF of the residual distribution. Normally distributed residuals would lie on the dotted line. The distributions are approximately normal for all groups. (JPG) [file pone.0163205.s011.jpg]

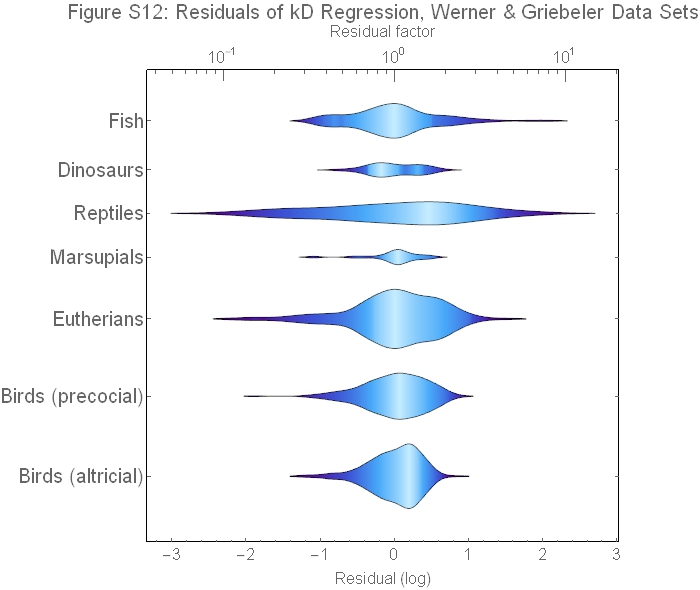

Supplement: S12 Fig — The height of each distribution is scaled proportionate to the square root of the number of samples. Multiple maxima for some groups indicates there may be some structure left in the residuals. (JPG) [file pone.0163205.s012.jpg]

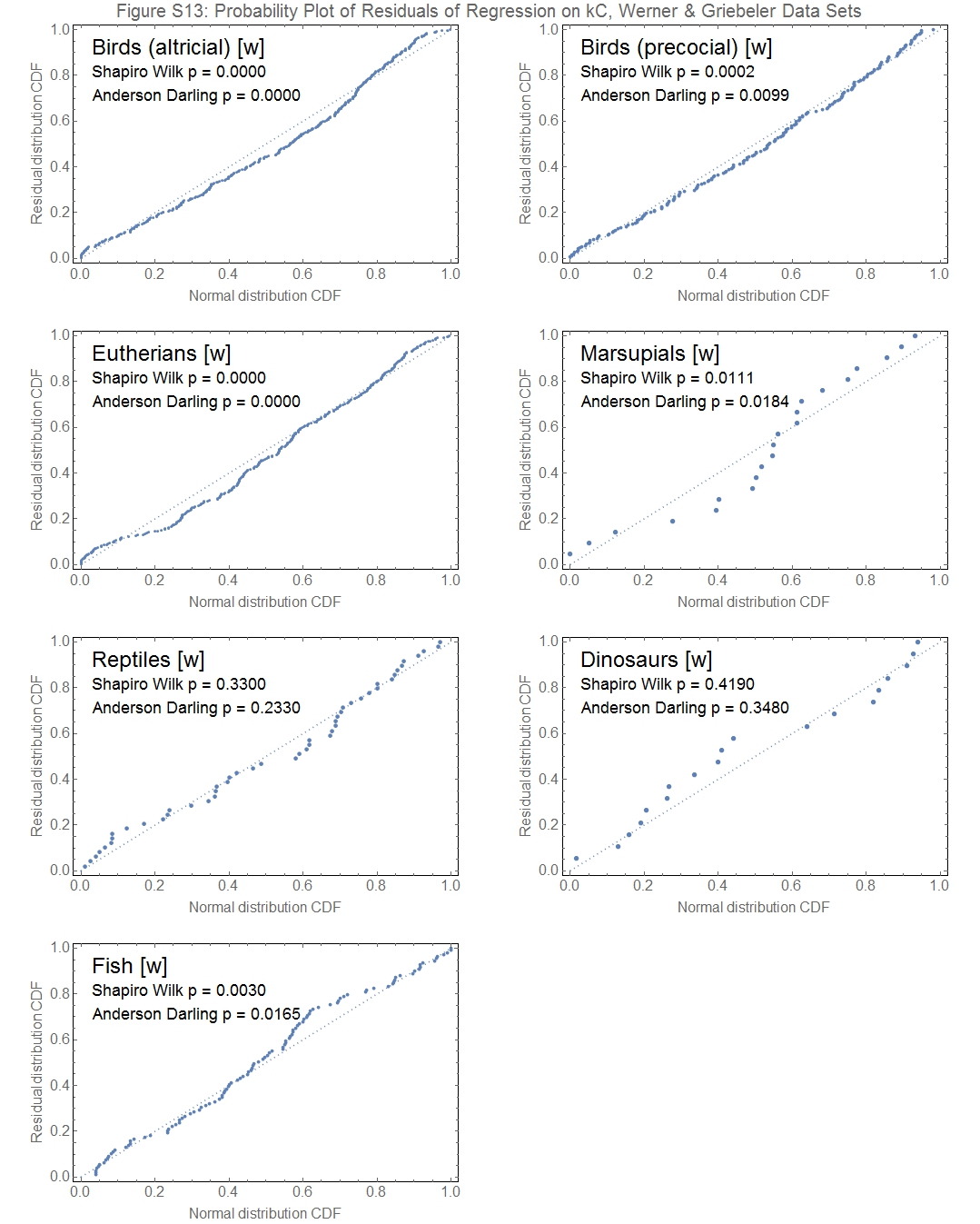

Supplement: S13 Fig — The x-axis is the cumulative probability distribution function (CDF) of the normal distribution, and the y-axis is the CDF of the residual distribution. Normally distributed residuals would lie on the dotted line. The distributions are approximately normal for all groups. (JPG) [file pone.0163205.s013.jpg]

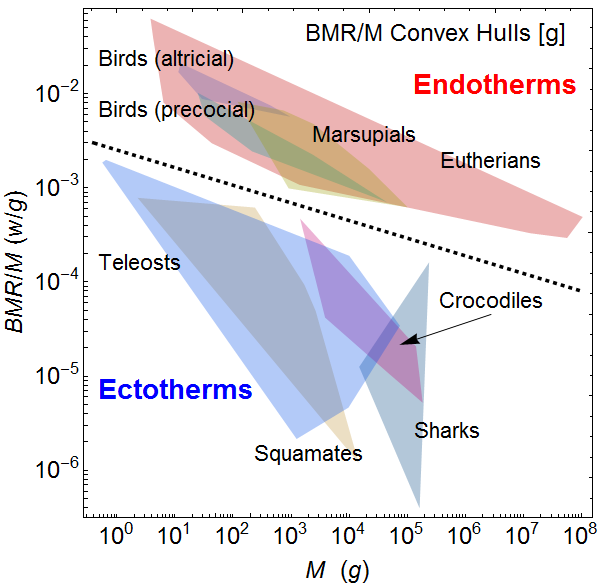

Supplement: S14 Fig — BMR versus M data from [13] was converted to BMR/M versus M; shaded regions are convex hulls. (PNG) [file pone.0163205.s014.png]

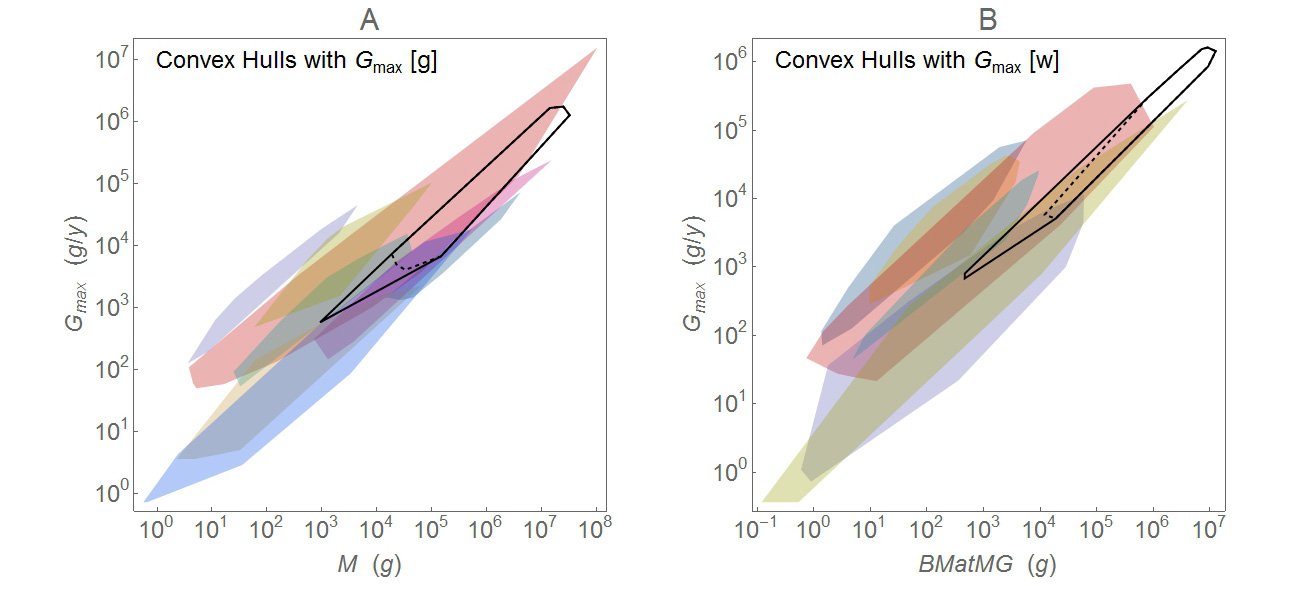

Supplement: S15 Fig — (A) plots data from Grady et al. [13], (B) from Werner and Griebeler [12]. This figure shows the range of variation using Gmax rather than kC, as used in Figs 5B and 4C. The groups are not labeled because they are too close together, but the color coding follows Fig 5 and S16 Fig. Dinosaurs are indicated by the black outline; dinosaurs without Archaeopteryx are shown by the dashed black line. (JPG) [file pone.0163205.s015.jpg]

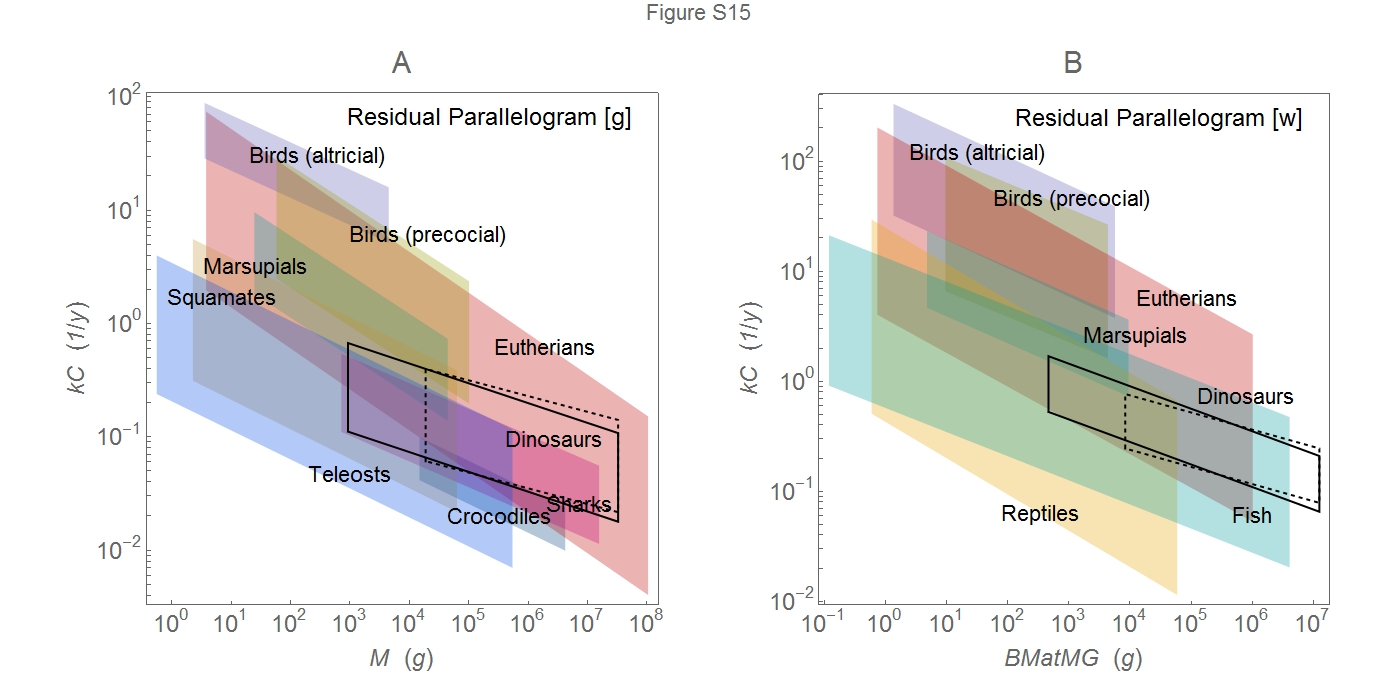

Supplement: S16 Fig — (A) plots data from Grady et al. [13], (B) from Werner and Griebeler [12]. Each parallelogram was formed by taking the regression line for the group; the top boundary is the regression line plus largest positive residual, and the bottom is the regression line plus the most negative residual. The horizontal extent of the parallelogram is determined by the range of the data. Dinosaurs are indicated by the black outline; dinosaurs without Archaeopteryx are shown with a dashed black line. (JPG) [file pone.0163205.s016.jpg]

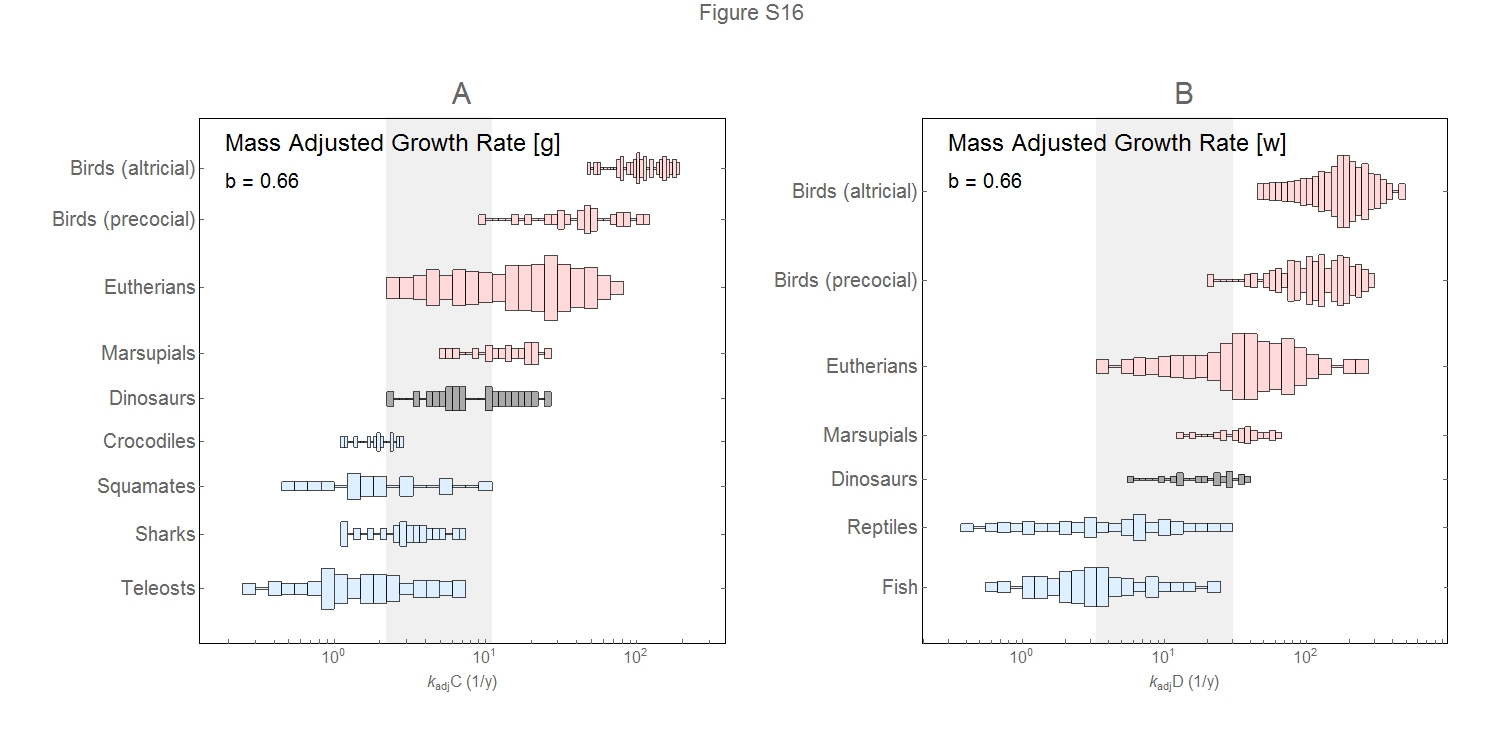

Supplement: S17 Fig — (A) plots data from Grady et al. [13], (B) from Werner and Griebeler [12]. This figure is the equivalent of Fig 6 but with a different value of the slope parameter b. (JPG) [file pone.0163205.s017.jpg]

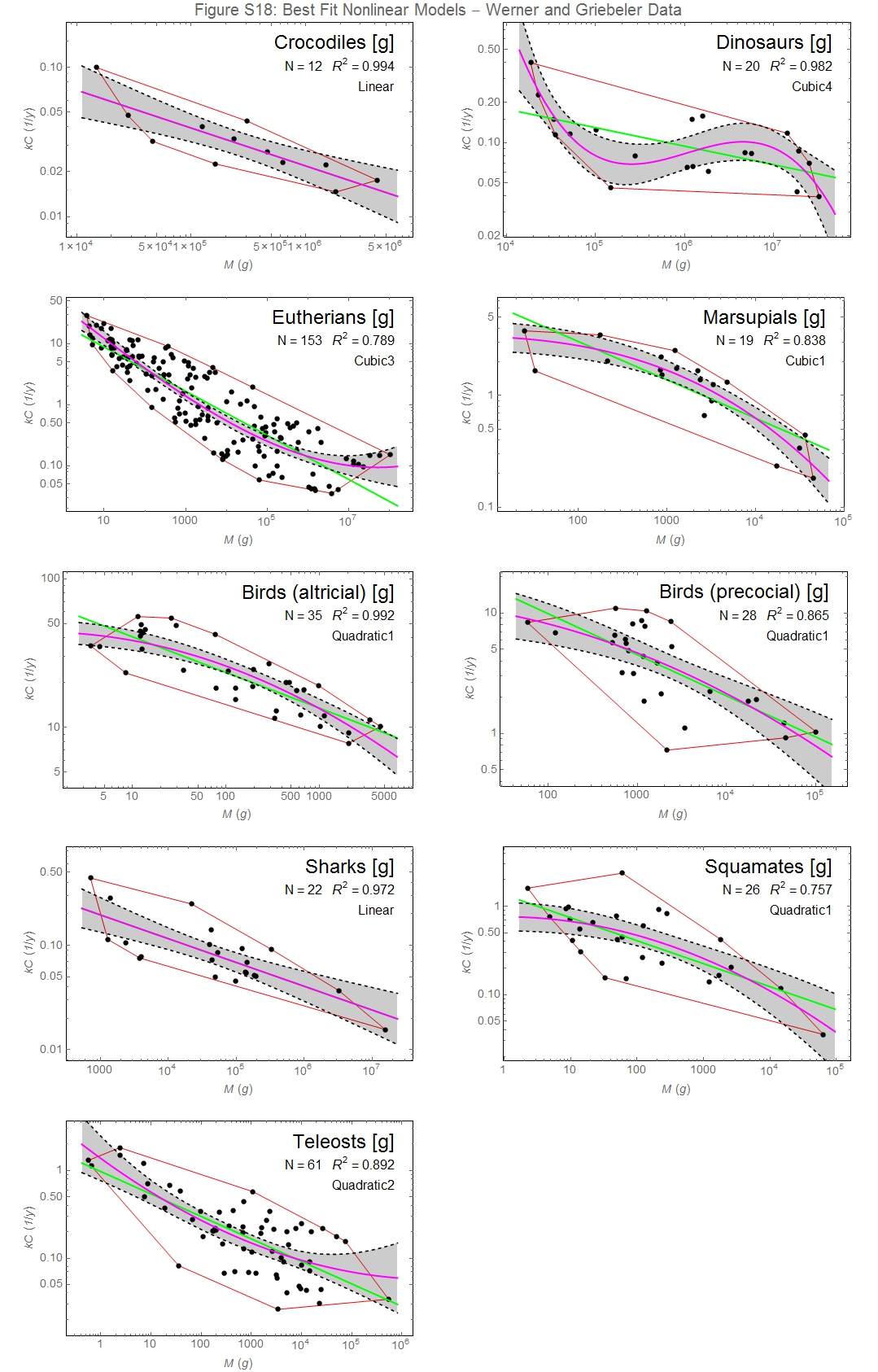

Supplement: S18 Fig — The best-fit model is plotted in magenta. The 95% confidence band is light gray and bordered by dashed lines. The best-fit linear model is plotted in green, except for groups where the best fit is already linear. See Table 3 for corresponding ΔAICc values. (JPG) [file pone.0163205.s018.jpg]

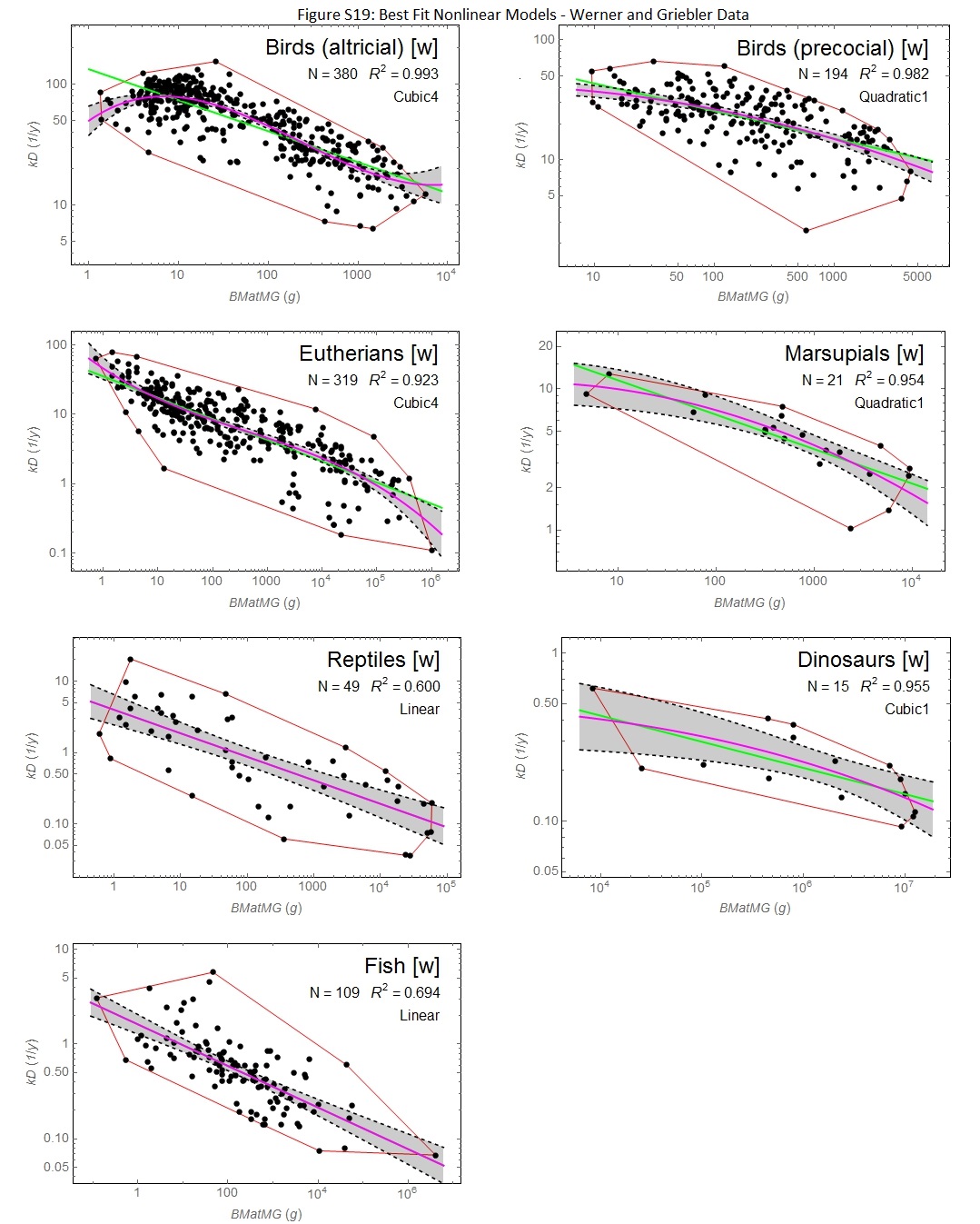

Supplement: S19 Fig — The best-fit model is plotted in magenta. The 95% confidence band is light gray and bordered by dashed lines. The best-fit linear model is plotted in green, except for groups where the best fit is already linear. See Table 3 for corresponding ΔAICc values. (JPG) [file pone.0163205.s019.jpg]

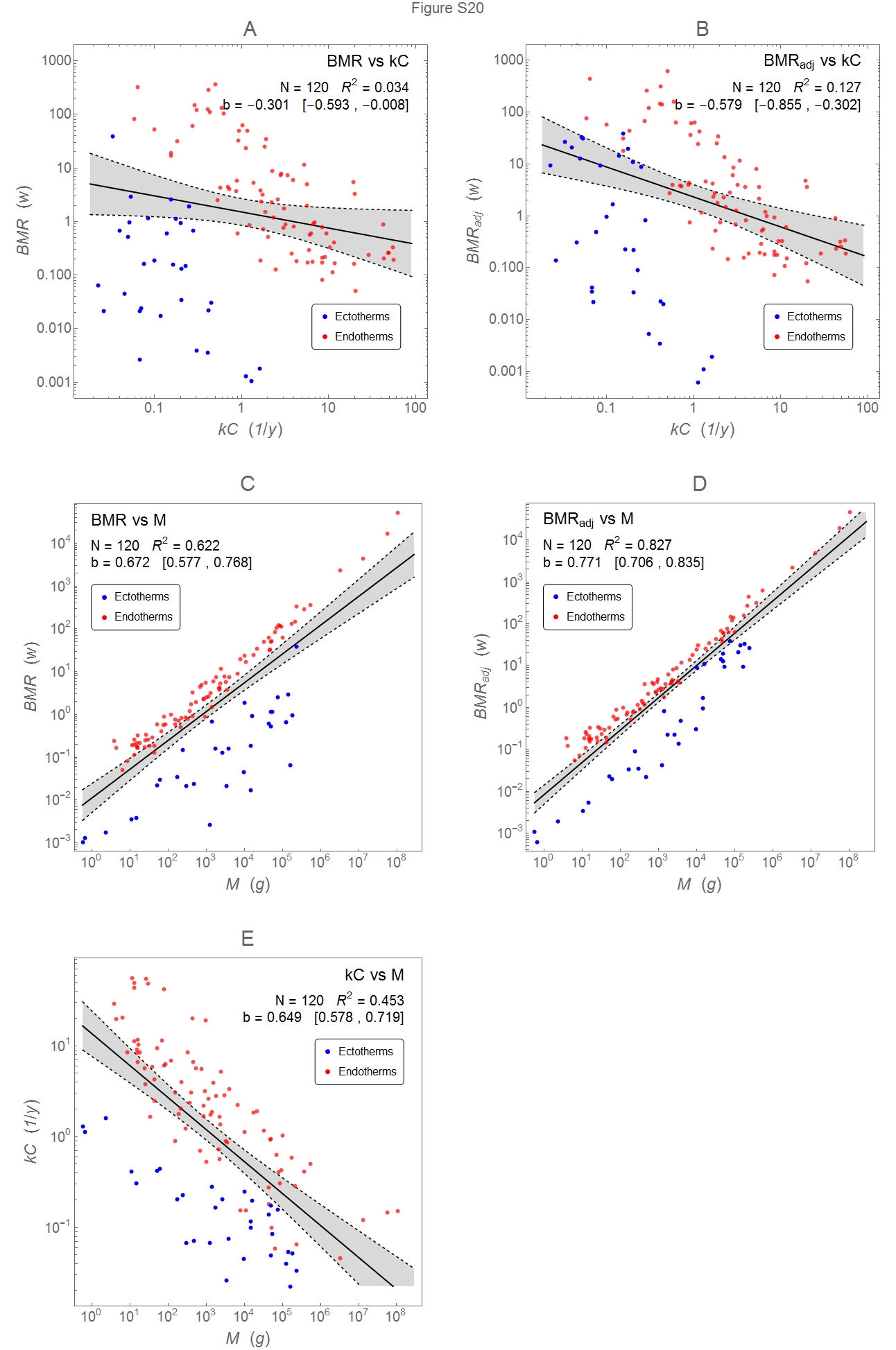

Supplement: S20 Fig — (A) plots pairwise correlation between BMR and kC, (B) between BMRadj and kC, (C) between BMR and M, (D) between BMRadj and M, and (E) between kC and M (see also S20 Fig). Even though both BMR and kC have correlations to M, they are essentially not correlated to each other (R2 = 0.03). The situation for BMRadj is similar. (JPG) [file pone.0163205.s020.jpg]

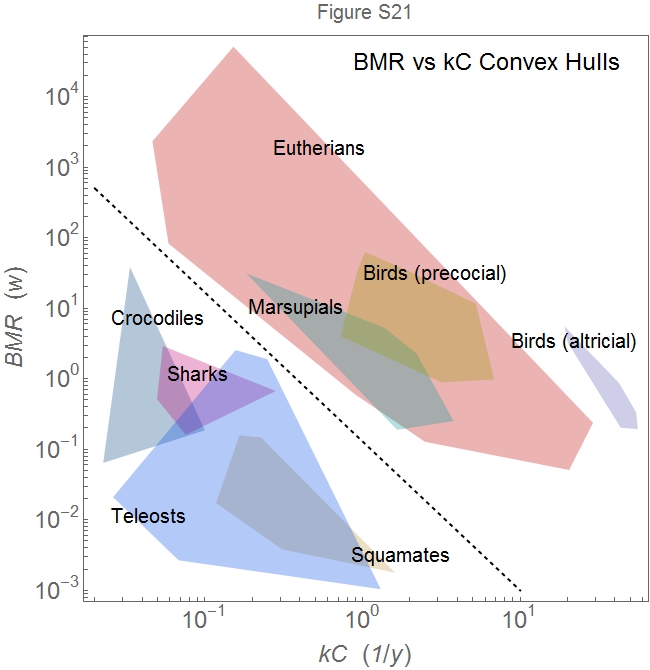

Supplement: S21 Fig — Using these variables, the extant groups from metabolic data of Grady et al. [13] separate cleanly into distinct endothermic and exothermic clusters. The dashed line separates the two groups. The situation is quite different than that shown in Fig 5, where ectotherms and endotherms overlap to a great extent. (JPG) [file pone.0163205.s021.jpg]

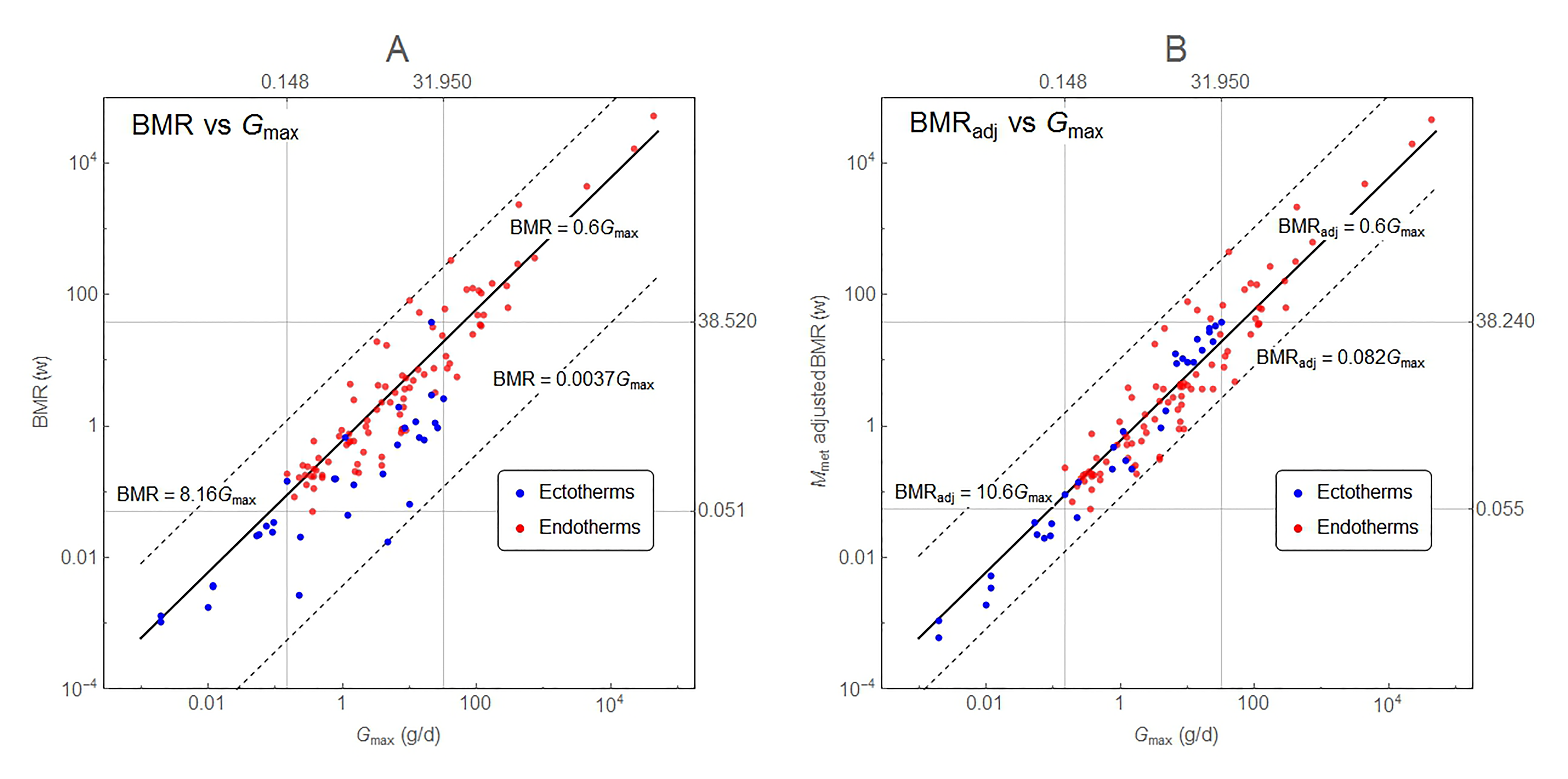

Supplement: S22 Fig — (A) Metabolic data from Grady et al. [13], is plotted on a log-log scale to test the hypothesis that = 0.6 Gmax. (B) the same relation is tested with BMRadj from Eq (19). Both BMR and Gmax are positively correlated with M, so we expect some relationship, but the error is very large, as shown by the dashed lines. BMR is underestimated by a factor of 13.6 and overestimated by a factor of 161; BMRadj does somewhat better, it is underestimated by a factor of 17.7 and overestimated by a factor of 7.3. The grid lines show the region where growth rates for endotherms and ectotherms overlap. (PNG) [file pone.0163205.s022.png]

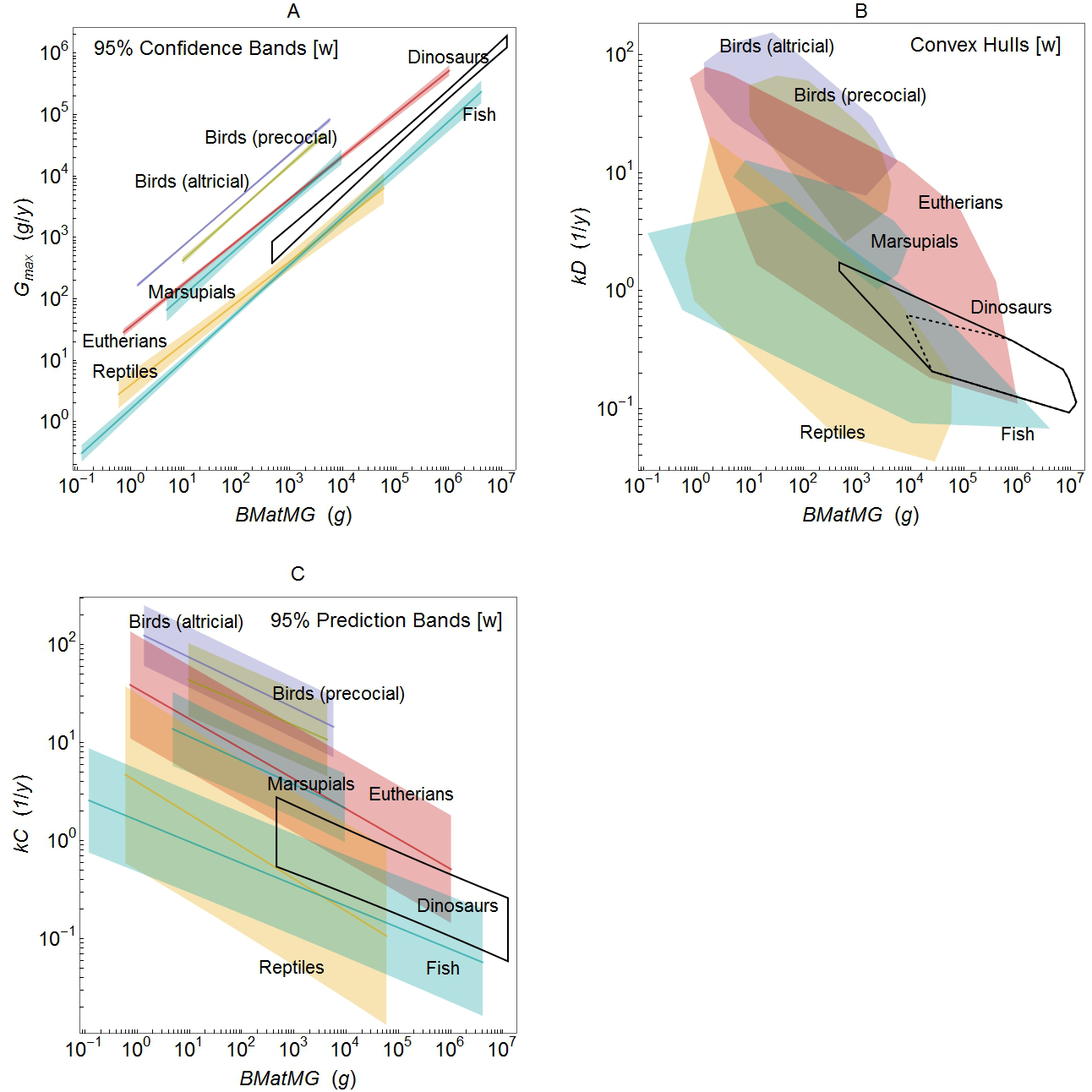

Supplement: S23 Fig — The equivalent of Fig 5 is plotted using the data sets from [12]. Note that there is no plot (D) because [12] does not include mass versus BMR data. (PNG) [file pone.0163205.s023.png]

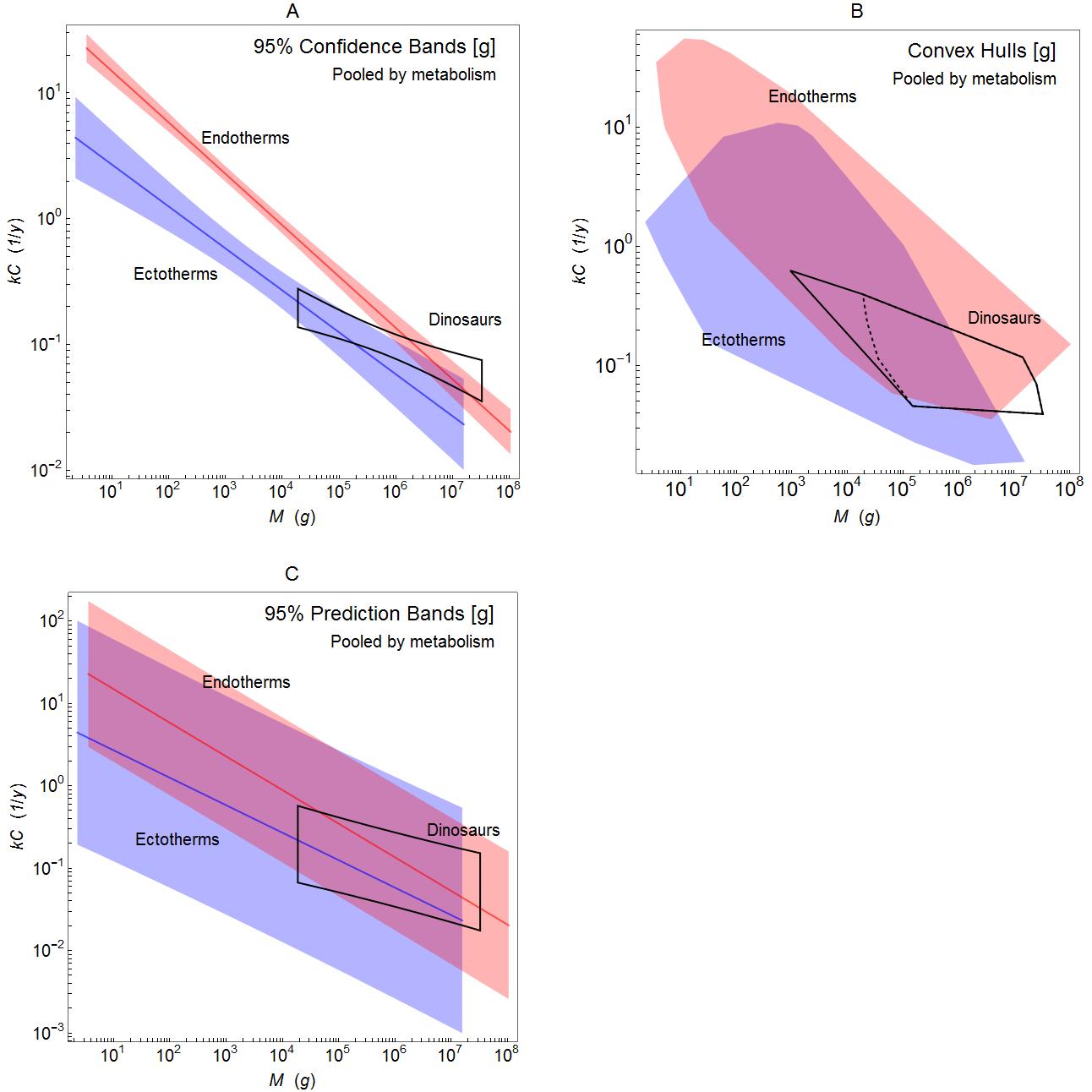

Supplement: S24 Fig — The equivalent of Fig 5 is plotted using data sets for extant animals from [13] after pooling the data into two groups, one containing all endotherms and another containing all ectotherms. (PNG) [file pone.0163205.s024.png]

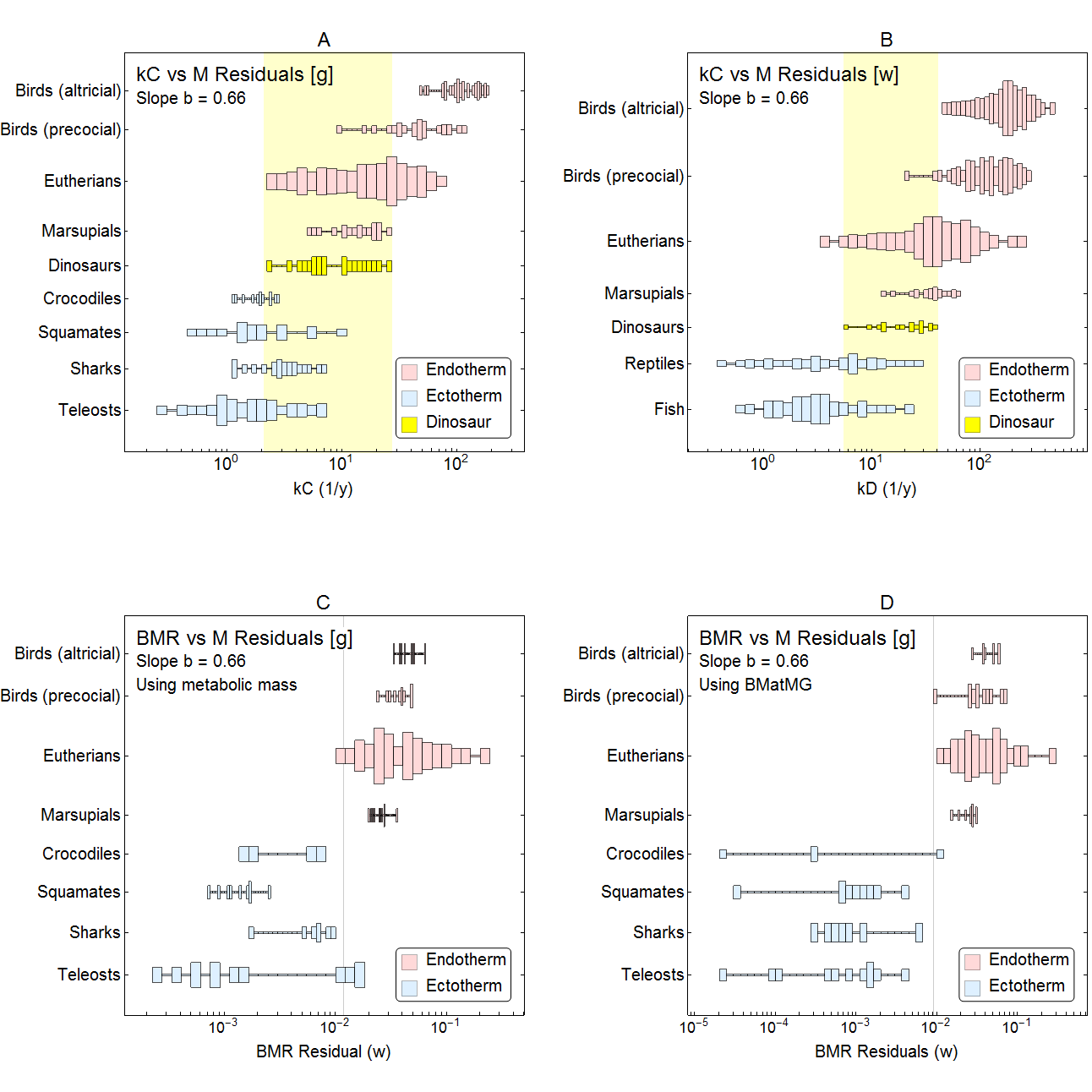

Supplement: S25 Fig — The equivalent of Fig 6 is plotted using a fixed slope b = 0.66. (PNG) [file pone.0163205.s025.png]
