# Supplementary material for: Dinosaur Metabolism and the Allometry of Maximum Growth Rate
Source: PLoS One. 2016 Nov 9;11(11):e0163205. doi: 10.1371/journal.pone.0163205 (PMC5102473; doi:10.1371/journal.pone.0163205)
Supplement: S1 Table — See S1 Text, S1–S3 Figs. (DOCX) [file pone.0163205.s026.docx]

**S1 Table.** **Corrected growth-rate data points for dinosaurs.** See S1 Text, S1-S3 Figs.

| **Grady et al. [13] Dinosaur Data Set** | | | | |
| --- | --- | --- | --- | --- |
|  | **Original** | | **Corrected** | |
| **Taxon** | $M$ **(g)** | $kC$ **(1/y)** | $M$ **(g)** | $kC$ **(1/y)** |
| *Psittacosaurus mongoliensis* | 22720 | 0.2219 | 22720 | 0.2282 |
| *Dysalotosaurus lettowvorbecki* | 148100 | 0.0458 | 148126 | 0.0458 |
| *Tenontosaurus tilletti* | 1084000 | 0.0655 | 1084020 | 0.0655 |
| *Massospondylus carinatus* | 281000 | 0.0987 | 281000 | 0.0795 |
| *Plateosaurus engelhardti* | 1587000 | 0.1590 | 1587000 | 0.1590 |
| *Alamosaurus sanjuanensis* | 32660000 | 0.0393 | 32663000 | 0.0393 |
| *Apatosaurus* | 19170000 | 0.0866 | 19165200 | 0.0866 |
| *Camarasaurus* | 14250000 | 0.1177 | 14247000 | 0.1177 |
| *Diplodocid* MfN.R.NW4 | 4753000 | 0.0838 | 18463000 | 0.0430 |
| *Diplodocid* MfN.R.2526 | 18460000 | 0.0430 | 4753000 | 0.0838 |
| Mamenchisaurid | 25080000 | 0.0704 | 25075000 | 0.0705 |
| *Allosaurus fragilis* | 1862000 | 0.0612 | 1861500 | 0.0612 |
| *Coelophysis bauri* | 33080 | 0.1239 | 35689.4 | 0.1144 |
| *Megapnosaurus rhodesiensis* | 18780 | 0.3946 | 18780 | 0.3977 |
| *Albertosaurus sarcophagus* | 1239000 | 0.1392 | 1223000 | 0.1496 |
| *Archaeopteryx lithographica* | 928 | 0.6297 | 928 | 0.6299 |
| *Citipati osmolskae* | 101700 | 0.1245 | 101722 | 0.1244 |
| *Gorgosaurus libratus* | 1733000 | 0.0476 | 1239000 | 0.0661 |
| *Saurornitholestes langstoni* | 34240 | 0.1506 | 34237.9 | 0.1506 |
| *Troodon formosus* | 52090 | 0.1164 | 52086.7 | 0.1164 |
| *Tyrannosaurus rex* | 5654000 | 0.0835 | 5654000 | 0.0835 |
| **Werner and Griebeler [12] Dinosaur Data Set** | | | | |
| **Taxon** | $BMatMG$ **(g)** | $kD$ **(1/y)** | $BMatMG$ **(g)** | $kD$ **(1/y)** |
| *Psittacosaurus lujiatunensis* | 18690 | 0.2734 | 25450 | 0.2063 |
| *Psittacosaurus mongoliensis* | 12000 | 0.4855 | 8358.22 | 0.6202 |
| *Alamosaurus* | 9481480 | 0.0997 | 12016000 | 0.1068 |
| *Alamosaurus* | 9481480 | 0.1150 |  |  |
| *Alamosaurus* | 9481480 | 0.1224 |  |  |
| *Apatosaurus* BYU601-17328 | 9089000 | 0.1777 | 9089000 | 0.1777 |
| *Apatosaurus* SMA0014 | 10103000 | 0.1455 | 10103000 | 0.1455 |
| *Camarasaurus* | 7123500 | 0.2148 | 7123500 | 0.2148 |
| *Mamenchisaurus* | 12537500 | 0.1138 | 12537500 | 0.1138 |
| *Diplodocid* MfN.R.2526 | 2376500 | 0.1385 | 2376500 | 0.1385 |
| *Diplodocid* MfN.R.NW4 | 9231500 | 0.0924 | 9231500 | 0.0924 |
| *Massospondylus carinatus* | 140000 | 0.2473 | 103374 | 0.2162 |
| *Plateosaurus* | 793500 | 0.3771 | 793500 | 0.3771 |
| *Plateosaurus* | 793500 | 0.3132 | 793500 | 0.3132 |
| *Albertosaurus sarcophagus* | 614000 | 0.1988 | 449917 | 0.4067 |
| *Archaeopteryx* | 464 | 1.4719 | 464.05 | 1.4719 |
| *Archaeopteryx* | 464 | 1.7316 | 464.05 | 1.7316 |
| *Gorgosaurus libratus* | 622000 | 0.2317 | 455803 | 0.1798 |
| *Tyrannosaurus rex* | 2780000 | 0.2761 | 2079990 | 0.2270 |
