# Supplementary material for: Dinosaur Metabolism and the Allometry of Maximum Growth Rate
Source: PLoS One. 2016 Nov 9;11(11):e0163205. doi: 10.1371/journal.pone.0163205 (PMC5102473; doi:10.1371/journal.pone.0163205)
Supplement: S2 Table — The independent variable x is either log(M) or log(BMatMG). (DOCX) [file pone.0163205.s027.docx]

**S2 Table.** **Models used for fitting growth rate to mass.** The independent variable $x$ is either $log\left(M\right)$ or $log\left(BMatMG\right)$.

| Function | Formula |
| --- | --- |
| Linear | $a+bx$ |
| Quadratic1 | $a+cx^{2}$ |
| Quadratic2 | $a+bx+cx^{2}$ |
| Cubic1 | $a+dx^{3}$ |
| Cubic2 | $a+cx^{2}+dx^{3}$ |
| Cubic3 | $a+bx+dx^{3}$ |
| Cubic4 | $a+bx+cx^{2}+dx^{3}$ |
