# Supplementary material for: Dinosaur Metabolism and the Allometry of Maximum Growth Rate
Source: PLoS One. 2016 Nov 9;11(11):e0163205. doi: 10.1371/journal.pone.0163205 (PMC5102473; doi:10.1371/journal.pone.0163205)
Supplement: S3 Table — Table 1 shows the data points from Grady et al.[13] arranged vertically, and the regression line associated with each group horizontally. Every data point is closest to a unique regression line. Table 2 shows data points from Werner and Griebeler [12]. (DOCX) [file pone.0163205.s028.docx]

**S3 Table.** **Data points closest to each regression line.** Table (A) shows the data points from Grady et al. [13] arranged vertically and the regression line associated with each group horizontally. Every data point is closest to a unique regression line. Table (B) shows data points from Werner and Griebeler [12].

(A)

|  |  | Data Points | | | | | | | | |
| --- | --- | --- | --- | --- | --- | --- | --- | --- | --- | --- |
|  |  | Birds (altricial) | Birds (precocial) | Eutherians | Marsupials | Dinosaurs | Crocodiles | Squamates | Sharks | Teleosts |
| Nearest Regression Line | Birds (altricial) | 32 | 6 | 0 | 0 | 0 | 0 | 0 | 0 | 0 |
|  | Birds (precocial) | 3 | 18 | 50 | 0 | 1 | 0 | 0 | 0 | 0 |
|  | Eutherians | 0 | 3 | 40 | 10 | 5 | 0 | 0 | 0 | 0 |
|  | Marsupials | 0 | 1 | 26 | 7 | 2 | 0 | 1 | 0 | 0 |
|  | Dinosaurs | 0 | 0 | 19 | 2 | 10 | 0 | 8 | 5 | 15 |
|  | Crocodiles | 0 | 0 | 0 | 0 | 0 | 9 | 5 | 5 | 28 |
|  | Squamates | 0 | 0 | 6 | 0 | 1 | 0 | 6 | 6 | 10 |
|  | Sharks | 0 | 0 | 11 | 0 | 1 | 1 | 3 | 4 | 2 |
|  | Teleosts | 0 | 0 | 1 | 0 | 1 | 2 | 3 | 2 | 6 |

(B)

|  | | Data Points | | | | | | |
| --- | --- | --- | --- | --- | --- | --- | --- | --- |
|  |  | Birds (altricial) | Birds (precocial) | Eutherians | Marsupials | Dinosaurs | Reptiles | Fish |
| Nearest regression line | Birds (altricial) | 294 | 66 | 1 | 0 | 0 | 0 | 0 |
|  | Birds (precocial) | 80 | 107 | 53 | 0 | 0 | 0 | 0 |
|  | Eutherians | 6 | 20 | 135 | 7 | 3 | 0 | 0 |
|  | Marsupials | 0 | 1 | 102 | 13 | 0 | 4 | 2 |
|  | Dinosaurs | 0 | 0 | 21 | 1 | 15 | 16 | 12 |
|  | Reptiles | 0 | 0 | 2 | 0 | 0 | 11 | 23 |
|  | Fish | 0 | 0 | 5 | 0 | 1 | 18 | 72 |
