# Supplementary material for: Dinosaur Metabolism and the Allometry of Maximum Growth Rate
Source: PLoS One. 2016 Nov 9;11(11):e0163205. doi: 10.1371/journal.pone.0163205 (PMC5102473; doi:10.1371/journal.pone.0163205)
Supplement: S4 Table — [g]: data from Grady et al. [13]; [w]: data from Werner and Griebeler [12]. (DOCX) [file pone.0163205.s029.docx]

**S4 Table.** **Table of regression results.** [g]: data from Grady et al. [13]; [w]: data from Werner and Griebeler [12].

| **Where** | **Data Set** | **Ind Var** | **Dep Var** | **N** | **R2** | **a** | **95%CI** | | **b** | **95% CI** | |
| --- | --- | --- | --- | --- | --- | --- | --- | --- | --- | --- | --- |
| Figs 3-5, S4-S9 Figs | Crocodiles [g] | *M* | *G*_max_ | 12 | 0.956 | -0.319 | -1.730 | 1.093 | 0.746 | 0.634 | 0.859 |
|  | Dinosaurs [g] | *M* | *G*_max_ | 21 | 0.963 | 0.126 | -0.935 | 1.188 | 0.821 | 0.744 | 0.899 |
|  | Eutherians [g] | *M* | *G*_max_ | 153 | 0.909 | 3.010 | 2.713 | 3.307 | 0.639 | 0.606 | 0.671 |
|  | Marsupials [g] | *M* | *G*_max_ | 19 | 0.898 | 2.692 | 1.820 | 3.565 | 0.657 | 0.543 | 0.770 |
|  | Birds (altricial) [g] | *M* | *G*_max_ | 35 | 0.965 | 4.260 | 3.991 | 4.528 | 0.760 | 0.708 | 0.811 |
|  | Birds (precocial) [g] | *M* | *G*_max_ | 28 | 0.798 | 3.854 | 2.822 | 4.887 | 0.660 | 0.526 | 0.793 |
|  | Sharks [g] | *M* | *G*_max_ | 22 | 0.948 | -0.058 | -0.988 | 0.871 | 0.772 | 0.688 | 0.857 |
|  | Squamates [g] | *M* | *G*_max_ | 26 | 0.890 | 0.302 | -0.272 | 0.877 | 0.740 | 0.630 | 0.849 |
|  | Teleosts [g] | *M* | *G*_max_ | 61 | 0.912 | -0.025 | -0.462 | 0.412 | 0.744 | 0.684 | 0.804 |
|  | Birds (altricial) [w] | *BMatMG* | *G*_max_ | 380 | 0.944 | 4.897 | 4.818 | 4.977 | 0.743 | 0.725 | 0.761 |
|  | Birds (precocial) [w] | *BMatMG* | *G*_max_ | 194 | 0.870 | 4.303 | 4.072 | 4.534 | 0.770 | 0.727 | 0.812 |
|  | Eutherians [w] | *BMatMG* | *G*_max_ | 319 | 0.922 | 3.566 | 3.429 | 3.704 | 0.694 | 0.671 | 0.716 |
|  | Marsupials [w] | *BMatMG* | *G*_max_ | 21 | 0.951 | 3.007 | 2.458 | 3.555 | 0.756 | 0.674 | 0.838 |
|  | Reptiles [w] | *BMatMG* | *G*_max_ | 49 | 0.856 | 1.380 | 0.889 | 1.870 | 0.671 | 0.590 | 0.751 |
|  | Dinosaurs [w] | *BMatMG* | *G*_max_ | 19 | 0.984 | 1.590 | 0.892 | 2.287 | 0.775 | 0.724 | 0.825 |
|  | Fish [w] | *BMatMG* | *G*_max_ | 109 | 0.931 | 0.479 | 0.241 | 0.718 | 0.781 | 0.740 | 0.821 |
| Figs 3 and 4, S4-S13 Figs | Crocodiles [g] | *M* | *kC* | 12 | 0.717 | -0.319 | -1.730 | 1.093 | -0.254 | -0.366 | -0.141 |
|  | Dinosaurs [g] | *M* | *kC* | 21 | 0.549 | 0.126 | -0.935 | 1.188 | -0.179 | -0.256 | -0.101 |
|  | Eutherians [g] | *M* | *kC* | 153 | 0.762 | 3.010 | 2.713 | 3.307 | -0.361 | -0.394 | -0.329 |
|  | Marsupials [g] | *M* | *kC* | 19 | 0.706 | 2.692 | 1.820 | 3.565 | -0.343 | -0.457 | -0.230 |
|  | Birds (altricial) [g] | *M* | *kC* | 35 | 0.731 | 4.260 | 3.991 | 4.528 | -0.240 | -0.292 | -0.189 |
|  | Birds (precocial) [g] | *M* | *kC* | 28 | 0.513 | 3.854 | 2.822 | 4.887 | -0.340 | -0.474 | -0.207 |
|  | Sharks [g] | *M* | *kC* | 22 | 0.613 | -0.058 | -0.988 | 0.871 | -0.228 | -0.312 | -0.143 |
|  | Squamates [g] | *M* | *kC* | 26 | 0.501 | 0.302 | -0.272 | 0.877 | -0.260 | -0.370 | -0.151 |
|  | Teleosts [g] | *M* | *kC* | 61 | 0.551 | -0.025 | -0.462 | 0.412 | -0.256 | -0.316 | -0.196 |
|  | Birds (altricial) [w] | *BMatMG* | *kD* | 380 | 0.668 | 4.897 | 4.818 | 4.977 | -0.257 | -0.275 | -0.239 |
|  | Birds (precocial) [w] | *BMatMG* | *kD* | 194 | 0.375 | 4.303 | 4.072 | 4.534 | -0.230 | -0.273 | -0.188 |
|  | Eutherians [w] | *BMatMG* | *kD* | 319 | 0.698 | 3.566 | 3.429 | 3.704 | -0.306 | -0.329 | -0.284 |
|  | Marsupials [w] | *BMatMG* | *kD* | 21 | 0.670 | 3.007 | 2.458 | 3.555 | -0.244 | -0.326 | -0.162 |
|  | Reptiles [w] | *BMatMG* | *kD* | 49 | 0.590 | 1.380 | 0.889 | 1.870 | -0.329 | -0.410 | -0.249 |
|  | Dinosaurs [w] | *BMatMG* | *kD* | 15 | 0.507 | 0.578 | -0.702 | 1.858 | -0.156 | -0.248 | -0.064 |
|  | Fish [w] | *BMatMG* | *kD* | 109 | 0.516 | 0.479 | 0.241 | 0.718 | -0.219 | -0.260 | -0.179 |
| Fig. 5 | Crocodiles [g] | *M* | *kC* | 12 | 0.994 | -0.364 | -0.538 | -0.189 |  |  |  |
|  | Dinosaurs [g] | *M* | *kC* | 21 | 0.953 | 1.084 | 0.847 | 1.321 |  |  |  |
|  | Eutherians [g] | *M* | *kC* | 153 | 0.690 | 2.108 | 1.951 | 2.265 |  |  |  |
|  | Marsupials [g] | *M* | *kC* | 19 | 0.664 | 1.999 | 1.754 | 2.244 |  |  |  |
|  | Birds (altricial) [g] | *M* | *kC* | 35 | 0.991 | 4.307 | 4.204 | 4.410 |  |  |  |
|  | Birds (precocial) [g] | *M* | *kC* | 28 | 0.855 | 3.174 | 2.946 | 3.402 |  |  |  |
|  | Sharks [g] | *M* | *kC* | 22 | 0.971 | 0.180 | -0.020 | 0.381 |  |  |  |
|  | Squamates [g] | *M* | *kC* | 26 | 0.746 | 0.255 | -0.011 | 0.522 |  |  |  |
|  | Teleosts [g] | *M* | *kC* | 61 | 0.887 | -0.066 | -0.240 | 0.108 |  |  |  |
|  | Birds (altricial) [w] | *BMatMG* | *kD* | 380 | 0.992 | 4.871 | 4.835 | 4.907 |  |  |  |
|  | Birds (precocial) [w] | *BMatMG* | *kD* | 194 | 0.981 | 4.407 | 4.346 | 4.467 |  |  |  |
|  | Eutherians [w] | *BMatMG* | *kD* | 319 | 0.915 | 3.268 | 3.195 | 3.340 |  |  |  |
|  | Marsupials [w] | *BMatMG* | *kD* | 21 | 0.952 | 3.045 | 2.884 | 3.206 |  |  |  |
|  | Reptiles [w] | *BMatMG* | *kD* | 49 | 0.566 | 0.988 | 0.693 | 1.284 |  |  |  |
|  | Dinosaurs [w] | *BMatMG* | *kD* | 19 | 0.950 | 1.913 | 1.730 | 2.095 |  |  |  |
|  | Fish [w] | *BMatMG* | *kD* | 109 | 0.688 | 0.638 | 0.524 | 0.752 |  |  |  |
| S18 Fig. | Crocodiles [g] | *M*_met_ | *BMR* | 4 | 0.941 | -6.450 | -7.542 | -5.358 |  |  |  |
|  | Eutherians [g] | *M*_met_ | *BMR* | 59 | 0.987 | -3.923 | -4.031 | -3.814 |  |  |  |
|  | Marsupials [g] | *M*_met_ | *BMR* | 10 | 0.994 | -4.294 | -4.390 | -4.198 |  |  |  |
|  | Birds (altricial) [g] | *M*_met_ | *BMR* | 8 | 0.989 | -3.448 | -3.576 | -3.321 |  |  |  |
|  | Birds (precocial) [g] | *M*_met_ | *BMR* | 11 | 0.984 | -4.010 | -4.231 | -3.789 |  |  |  |
|  | Sharks [g] | *M*_met_ | *BMR* | 7 | 0.683 | -5.778 | -6.269 | -5.287 |  |  |  |
|  | Squamates [g] | *M*_met_ | *BMR* | 9 | 0.994 | -6.996 | -7.249 | -6.743 |  |  |  |
|  | Teleosts [g] | *M*_met_ | *BMR* | 12 | 0.870 | -6.752 | -7.711 | -5.792 |  |  |  |
| S19 Fig. | Crocodiles [g] | *M* | *BMR* | 4 | 0.209 | -8.867 | -12.858 | -4.877 |  |  |  |
|  | Eutherians [g] | *M* | *BMR* | 59 | 0.984 | -3.953 | -4.072 | -3.833 |  |  |  |
|  | Marsupials [g] | *M* | *BMR* | 10 | 0.980 | -4.288 | -4.465 | -4.110 |  |  |  |
|  | Birds (altricial) [g] | *M* | *BMR* | 8 | 0.982 | -3.480 | -3.641 | -3.319 |  |  |  |
|  | Birds (precocial) [g] | *M* | *BMR* | 11 | 0.955 | -4.138 | -4.511 | -3.764 |  |  |  |
|  | Sharks [g] | *M* | *BMR* | 7 | -0.273 | -8.017 | -9.001 | -7.032 |  |  |  |
|  | Squamates [g] | *M* | *BMR* | 9 | 0.885 | -7.541 | -8.697 | -6.385 |  |  |  |
|  | Teleosts [g] | *M* | *BMR* | 12 | 0.853 | -8.055 | -9.072 | -7.038 |  |  |  |
| Fig. 6, S22-S26 Figs. | Metabolism data set | *G*_max_ | *BMR* | 120 | 0.843 | -1.065 | -1.324 | -0.805 | 1.036 | 0.954 | 1.118 |
|  | Metabolism data set | *kC* | *BMR* | 120 | 0.034 | 0.414 | -0.162 | 0.989 | -0.301 | -0.593 | -0.008 |
|  | Metabolism data set | *M* | *kC* | 120 | 0.453 | 2.603 | 2.028 | 3.179 | -0.351 | -0.422 | -0.281 |
|  | Metabolism data set | *M*_met_ | *BMR* | 120 | 0.836 | -5.195 | -5.705 | -4.685 | 0.833 | 0.766 | 0.901 |
|  | Metabolism data set | *M* | *BMR* | 120 | 0.622 | -4.481 | -5.262 | -3.701 | 0.672 | 0.577 | 0.768 |
