# Supplementary material for: Dinosaur Metabolism and the Allometry of Maximum Growth Rate
Source: PLoS One. 2016 Nov 9;11(11):e0163205. doi: 10.1371/journal.pone.0163205 (PMC5102473; doi:10.1371/journal.pone.0163205)
Supplement: S5 Table — The models are found in S2 Table, and the identification of the best-fit models is found in Table 3. [g]: data from Grady et al. [13]; [w]: data from Werner and Griebeler [12]. (DOCX) [file pone.0163205.s030.docx]

**S5 Table. Curvilinear regression results.** The models are found in S2 Table, and the identification of the best fit models is found in Table 2. [g]: data from Grady et al. [13]; [w]: data from Werner and Griebeler [12].

| **Data Set** | **IV** | **DV** | **N** | $R^{2}$ | **a** | **95% CI** | | **b** | **95% CI** | | **c** | **95% CI** | | **d** | **95% CI** | |
| --- | --- | --- | --- | --- | --- | --- | --- | --- | --- | --- | --- | --- | --- | --- | --- | --- |
| Crocodiles [g] | *M* | *kC* | 12 | 0.994 | -0.254 | -0.366 | -0.141 | -0.319 | -1.730 | 1.093 |  |  |  |  |  |  |
| Dinosaurs [g] | *M* | *kC* | 20 | 0.982 | -0.033 | -0.053 | -0.012 | 1.356 | 0.527 | 2.186 | -18.627 | -29.763 | -7.491 | 81.852 | 32.90 | 130.805 |
| Eutherians [g] | *M* | *kC* | 153 | 0.789 | 0.001 | 0.000 | 0.001 | -0.510 | -0.584 | -0.436 | 3.676 | 3.265 | 4.087 |  |  |  |
| Marsupials [g] | *M* | *kC* | 19 | 0.838 | -0.002 | -0.003 | -0.002 | 1.239 | 0.933 | 1.544 |  |  |  |  |  |  |
| Birds (altricial) [g] | *M* | *kC* | 35 | 0.992 | -0.025 | -0.030 | -0.020 | 3.782 | 3.608 | 3.956 |  |  |  |  |  |  |
| Birds (precocial) [g] | *M* | *kC* | 28 | 0.865 | -0.021 | -0.029 | -0.013 | 2.538 | 2.000 | 3.076 |  |  |  |  |  |  |
| Sharks [g] | *M* | *kC* | 22 | 0.972 | -0.228 | -0.312 | -0.143 | -0.058 | -0.988 | 0.871 |  |  |  |  |  |  |
| Squamates [g] | *M* | *kC* | 26 | 0.757 | -0.023 | -0.032 | -0.014 | -0.273 | -0.640 | 0.095 |  |  |  |  |  |  |
| Teleosts [g] | *M* | *kC* | 61 | 0.892 | 0.014 | -0.002 | 0.029 | -0.415 | -0.609 | -0.222 | 0.319 | -0.267 | 0.904 |  |  |  |
| Birds (altricial) [w] | *BMatMG* | *kC* | 380 | 0.993 | 0.010 | 0.006 | 0.015 | -0.165 | -0.223 | -0.108 | 0.523 | 0.289 | 0.758 | 3.908 | 3.628 | 4.188 |
| Birds (precocial) [w] | *BMatMG* | *kC* | 194 | 0.982 | -0.022 | -0.025 | -0.018 | 3.733 | 3.603 | 3.864 |  |  |  |  |  |  |
| Eutherians [w] | *BMatMG* | *kC* | 319 | 0.923 | -0.002 | -0.004 | 0.000 | 0.043 | 0.005 | 0.082 | -0.522 | -0.743 | -0.300 | 3.832 | 3.466 | 4.197 |
| Marsupials [w] | *BMatMG* | *kC* | 21 | 0.954 | -0.022 | -0.029 | -0.015 | 2.414 | 2.062 | 2.767 |  |  |  |  |  |  |
| Reptiles [w] | *BMatMG* | *kC* | 49 | 0.600 | -0.329 | -0.410 | -0.249 | 1.380 | 0.889 | 1.870 |  |  |  |  |  |  |
| Dinosaurs [w] | *BMatMG* | *kC* | 15 | 0.955 | 0.000 | 0.000 | 0.000 | -0.659 | -1.221 | -0.096 |  |  |  |  |  |  |
| Fish [w] | *BMatMG* | *kC* | 109 | 0.694 | -0.219 | -0.260 | -0.179 | 0.479 | 0.241 | 0.718 |  |  |  |  |  |  |
