# Supplementary material for: Dinosaur Metabolism and the Allometry of Maximum Growth Rate
Source: PLoS One. 2016 Nov 9;11(11):e0163205. doi: 10.1371/journal.pone.0163205 (PMC5102473; doi:10.1371/journal.pone.0163205)
Supplement: S1 Text — (DOCX) [file pone.0163205.s031.docx]

## S1 Text – Data Errors in Grady et al. [13] and Werner and Griebeler [12]

**Data issues with *Massospondylus* and *Megapnosaurus***

As described in S1 Text of ref. [14], the plots of *Massospondylus* and *Megapnosaurus* data used by Grady et al. [13] are incorrect. They do not plot the originally published data for these taxa. In the case of *Massospondylus*, the original data set assembled by Chinsamy-Turan [78] contains 17 data points. Erickson et al. [7] plotted 9 points, ostensibly from this data set, but of those 9, only 3 points matched the original data set.

A similar situation exists for *Megapnosaurus* (formerly *Syntarsus*). The plot used for the digitization by Grady et al. has six data points, whereas the original data set contains 10 data points. Only two of the plotted data points correspond to points in the original data set. In both cases, the plotted data sets do not match the original histological reference study and should not be used.

In the corrected Grady data set, the appropriate age–mass data sets are used for each taxon. I performed the same regression on them as Grady et al. did to obtain new mass–growth rate data for each taxon.

Werner and Griebeler [12] included *Massospondylus* but not *Megapnosaurus* in their analysis*.* Because Werner and Griebeler did not perform growth-data fits themselves, I instead used the age–growth rate data for *Massospondylus* from the corrected growth-data series from Grady et al.

**Data issues with Dolnik neonate formula**

Grady et al. used a formula by Dolnik [79,80] on dinosaur egg size to set the neonate size for dinosaur taxa in cases where no neonate specimens are available (i.e., most taxa). The formula is$m_{\text{neonate}}=1.459 M^{0.46}$ , where $m_{\text{neonate}}$ is the neonate mass in grams and $M$ is the adult mass in grams. In order to verify the data sets, I plotted the neonate masses and found that several of them were incorrect due to clerical errors (S1 Fig).

It should be noted that the data on which the Dolnik formula is based are quite sparse. As a result, adding hypothetical data points with this mass is a potential source of error.

**Data issues with *Archaeopteryx lithographica*, *Citipati osmolskae*, and *Troodon formosus***

The data for *Archaeopteryx lithographica*, *Citipati osmolskae*, and *Troodon formosus* come from digitized graphs prepared by Grady et al. from two papers by Erickson et al. [6,41]. Werner and Griebeler referenced the same studies.

In three papers by Erickson et al. published between 2001 and 2009 [6,7,11], one or more of the graphs exhibit serious problems as described in ref. [14], including plotting a data set other than the original (as happened with *Megapnosaurus* and *Massospondylus*).

Unfortunately, data are not available to check the 2007 and 2009 papers involving *Archaeopteryx lithographica*, *Citipati osmolskae*, and *Troodon formosus.* However, the problems observed in graphs in every case so far where replication has been attempted raises concerns about the accuracy of the charts for those taxa as well.

Because eggs are known for *Citipati osmolskae*, and *Troodon formosus*, neonate mass can be estimated directly; there is no need to rely on the Dolnik formula. However, due to a calculation error, the Grady et al. data set assigned far too low a value for neonate masses of these taxa—25 grams for Citipati and 31 grams for Troodon. In the corrected data, the values are 396 grams and 220 grams respectively (corresponding to 70% of presumed egg mass).

**Data issues with *Alamosaurus* in Werner and Griebeler [12]**

Werner and Griebeler [12] provide three data points for *Alamosaurus*: specimens A, B, and C. They reference Griebeler [81] as the source, which in turn references Lehman and Woodward [9]. However, the Lehman and Woodward paper lists only one specimen of *Alamosaurus*. The three curves presented in the paper are different *scenarios*, described as follows [9]:

In the case of *Alamosaurus*, a value of 0.075 for the exponent in the growth equation results in a reasonable fit to the data, with the first pre- served growth line produced at age four, and the outermost at age 12 (Fig. 2). Values for b less than 0.065 result in underestimation of mass, whereas values greater than 0.08 result in substantial overestimation at the upper end of the growth series (Table 2).

In the caption of the figure that plots the growth curve, the authors further note:

Growth annuli preserved on the lateral surface of a subadult *Alamosaurus* humerus, redrawn from a photograph given by Woodward (2005).

. . .

The growth history in terms of body mass (W) versus age (t) for *Alamosaurus* is based on fitting estimated masses for nine yearly growth increments (Tables 1 and 2) to the von Bertalanffy growth equation, and varying exponent (b) between 0.065 and 0.08.

Using multiple values from one specimen seems inappropriate for this purpose. Moreover, Lehman and Woodward caution against least-squares regressions on these data:

Because there remains substantial disagreement regarding the accuracy of methods for estimating mass in dinosaurs (e.g., Paul 1997), and considerable uncertainty regarding the assumptions used in this analysis (see discussion), a high degree of precision in curve fitting is unwarranted.

In view of these issues, I included a single *Alamosaurus* data point from the Grady et al. data set for my corrected data set.

**Concerns regarding metabolic data**

Grady et al. included basal metabolic rate (BMR) data for a subset of the extant species for which growth data are available. The BMR were measured at a specific mass, termed “metabolic mass” by Grady et al. Unfortunately, many studies make use of juvenile or very old specimens whose metabolic rates may be quite different than they are at the time of maximum growth rate. For *Crocodylus niloticus*, for example, the $BMatMG=162700g,$ but $MM=215.3g$. Measurement of the BMR at 0.1% of the mass at which maximum growth rate occurs, when the specimen is clearly juvenile, may be problematic. Because $BMatMG=M/e$, it is an even smaller fraction of adult mass.

Altogether, 22 of the 120 BMR data points occur at less than 50% of $BMatMG$, and 12 points occur at more than 150% of $BMatMG$. This amounts to 28% of the data points. Absent an obvious way to correct for this problem, data were used as is.

**Summary of data corrections made**

In the course of checking this work, the following data errors were found and corrected in the Grady et al. data sets:

1. Neonate mass for *Citipati* as discussed above.
2. Neonate mass for *Troodon* as discussed above.
3. Neonate mass for *Alamosaurus* was 4074 grams, more than twice the Dolnik formula at 2015 grams. This was corrected to the Dolnik value.
4. Neonate mass for *Coelophysis* was 216 grams, about 1.4X the Dolnik value. It was corrected to 151 grams.
5. The value $M_{\text{lit}}=1733$ kg was used for *Gorgosaurus*. The original reference was 1239 kg, so it was corrected to that value.
6. The value $M_{\text{lit}}=1239$ kg was used for *Albertosaurus*. The original reference was 1223 kg, so it was corrected to that value.
7. The value $M_{\text{lit}}=18463$ kg was used for one *Diplodocus* taxon and $M_{\text{lit}}=4753$ kg for a second distinct *Diplodocus* taxon. The correct values are reversed with respect to the data sets.
8. The incorrect data set was used for *Massospondylus* (see above). This was corrected.
9. The incorrect data set was used for *Megapnosaurus* (see above). This was corrected.
10. The Grady et al. data set for *Gorgosaurus* showed some small digitization errors, so the correct numerical data was substituted.
11. The Grady et al. data set for *Psittacosaurus* showed some small digitization errors, so the correct numerical data was substituted.

In each case the goal was to use the same procedures and approach as Grady et al. intended but to correct clerical data errors.

In the course of checking this work, the following data errors were found and corrected in the Werner and Griebeler data sets:

1. As discussed above, the incorrect data set was used for *Massospondylus*. The mass–growth rate data point was taken from that used for Grady et al.
2. *Alamosaurus* was reduced to a single data point, taken from Grady et al.
3. *Tyrannosaurus*, *Albertosaurus,* and *Gorgosaurus* data points were taken from Erickson et al. 2004 [5]. Because those data points cannot be replicated [14], the values from Grady et al. were used instead.
4. *Psittacosaurus mongoliensis* data was taken from Erickson et al. [7]. Because that growth rate cannot be replicated, the value from Grady et al. was used instead.
5. *Psittacosaurus lujiatunensis* data was taken from Erickson et al. [11]. Because that growth model cannot be replicated [14,15], the value found in ref. [14] was used instead.

In all cases when data were taken from another source, they were adjusted to use $BMatMG$ rather than $M$, in the same manner that Werner and Griebeler did.

**Additional References for S1 Text**

79. Dolnik VR. Body weight, energy metabolism, and time in the life of birds. Entomol Rev. 2006;86: S119–S127. doi:10.1134/S0013873806110017

80. Dolnik VR. Allometry of reproduction in poikilotherm and homoiotherm vertebrates. Biol Bull Russ Acad Sci. 2000;27: 591–600. Available: http://link.springer.com/article/10.1023/A:1026663632446

81. Griebeler EM. Body temperatures in dinosaurs: what can growth curves tell us? PLoS One. 2013;8: e74317. doi:10.1371/journal.pone.0074317
